# Impact of KRAS G12C mutation on the efficacy of chemoradiotherapy in patients with unresectable stage II or III non-small cell lung cancer

**DOI:** 10.3389/fonc.2025.1675825

**Published:** 2025-11-27

**Authors:** Jean Cabon, Delphine Lerouge, Sebastien Thureau, Jacques Balosso, Marin Guigo, Radj Gervais, Catherine Dubos, Pascal Dô, Pierre Demontrond, Hubert Curcio, Simon Deshayes, Jeannick Madelaine, Dimitri Leite Ferreira, Leonard Jacson, Kilian Lecrosnier, François Chevalier, Florian Guisier, Edouard Dantoing, Alexandra Leconte, François Christy, Mathieu Césaire

**Affiliations:** 1 Department of Medical Oncology, Centre François Baclesse, Caen, France; 2 Department of Radiation Oncology, Centre François Baclesse, Caen, France; 3 Department of Radiation Oncology, Centre Henri Becquerel, Rouen, France; 4 Department of Pneumology, Centre Hospitalier Universitaire de Caen, Caen, France; 5 UMR6252 CIMAP (Research Center for Ions, Materials, and Photonics), Team Applications in Radiobiology with Accelerated Ions, CEA-CNRS-ENSICAEN-Université de Caen Normandie, Caen, France; 6 Department of Pneumology, Centre Hospitalier Universitaire de Rouen, Rouen, France; 7 Department of Clinical Research, Centre François Baclesse, Caen, France

**Keywords:** non-small cell lung cancer, KRAS mutant, KRAS G12C mutation, chemoradiotherapy, locally advance disease

## Abstract

**Background:**

Approximately 35% of patients with non-small cell lung cancer (NSCLC) have locally advanced disease. Despite treatment with chemoradiotherapy (CRT) and consolidation immunotherapy, overall survival remains below 50% at 5 years. Kirsten rat sarcoma (*KRAS*) mutations (*KRAS*ms) are the most common lung cancer mutations, affecting 25% of NSCLC cases. *KRAS*m can cause radioresistance, and several targeted *KRASm* therapies have been developed, mainly targeting the *KRAS* G12C mutation (*KRASm* G12C), but the impact of *KRASm* G12C on the efficacy of CRT for locally advanced NSCLC remains unclear.

**Methods:**

We conducted a multicenter retrospective study of unresectable stage II or III NSCLC treated with CRT in four French hospitals between January 2014 and December 2022. The primary endpoint was the objective response rate (ORR) for *KRASm* G12C compared to *KRAS* wild-type (*KRASwt*). The main secondary objectives were to assess the difference in ORR between *KRASm* and *KRASwt*, and the difference in disease control rate (DCR), overall survival (OS), progression-free survival (PFS), time to local relapse (TTLR), and time to distant relapse (TTDR) according to *KRASm* status.

**Results:**

Our study included 267 patients, and 73 patients had *KRASm* (27.3%). The most common *KRASm* was *KRASm* G12C (n = 42). Tumors were lung adenocarcinoma in 91% (n = 244) of patients. Two hundred (75%) patients were treated with concomitant CRT. There was no difference between *KRASm* G12C and *KRASwt* patients in terms of ORR (48% vs. 49%; p = 0.961) and DCR (86% vs. 84%; p = 0.903), nor when comparing *KRASm* to *KRASwt* in terms of OS (p = 0.64), PFS (p = 0.28), TTLR (p = 0.26), and TTDR (p = 0.3), with no impact after adjustment for durvalumab.

**Conclusion:**

*KRAS* G12C mutation compared to KRAS wild-type did not affect response to chemoradiotherapy, and *KRAS* mutations compared to *KRAS* wild-type were not associated with worse survival in unresectable stage II or III NSCLC treated with chemoradiotherapy.

## Highlights

- *KRASm*s are the most common lung cancer oncogene mutations in NSCLC (25% of NSCLC cases), with a predominance of KRASm G12C in approximately 40%.
- *KRAS* G12C mutation, compared to *KRAS* wild-type, did not affect response to CRT.
- *KRAS* mutations compared to *KRAS* wild-type were not associated with worse survival in unresectable stage II or III NSCLC treated with CRT.

## Introduction

Lung cancer is a real public health problem worldwide. It is estimated to be the leading cause of cancer-related death, with 1.8 million deaths by 2020 (1). Non-small cell lung cancer (NSCLC) accounts for more than 80% of lung cancer cases, and approximately 35% of patients with NSCLC have locally advanced disease. Despite treatment with chemoradiotherapy (CRT) and consolidation immunotherapy, overall survival remains below 50% at 5 years in patients with unresectable stage III NSCLC (2). Lung cancer is a molecularly heterogeneous disease, and understanding its biology is crucial for the development of effective therapies (3).

Kirsten rat sarcoma (*KRAS*) mutations (*KRASm*s) are the most frequent lung cancer oncogenic mutations (25% of NSCLC and 30%–37% of adenocarcinoma cases), with the majority of cases involving codons 12 and 13 (4). Ras proteins are encoded by three ubiquitously expressed genes: *HRAS*, *KRAS*, and *NRAS*. *KRAS* is the most frequently mutated, followed by *NRAS* (5). These proteins are GTPases that function as molecular switches regulating pathways, responsible for cell proliferation and survival. Aberrant Ras function is associated with hyperproliferative developmental disorders and cancer (6). Among *KRASm*s in NSCLC, the *KRAS* G12C mutation (*KRASm* G12C) is the most common (approximately 40%), followed by the *KRAS* G12V mutation (20%) and the *KRAS* G12D mutation (16%) (7, 8). *KRASm*s are associated with smoking history and Caucasian ethnicity, with a higher proportion of never smokers for *KRAS* G12D compared to *KRASm* G12C and *KRAS* G12V mutations (9, 10).

Several studies have reported a negative impact of *KRASm* G12C on survival, but the prognostic impact remains controversial, with conflicting data in the literature (11–14).

In addition, *KRASm* may cause radioresistance, although the exact mechanisms remain unknown (15–18). In a retrospective study of stage III NSCLC treated with CRT, *KRASm*s were associated with a lower response rate (63% vs. 81%), which may indicate a reduced efficacy of CRT (19). However, the study included 119 patients with only 13% *KRASm*s and did not specify the subtype of *KRASm.*

Several targeted therapies have been developed, mainly targeting the *KRASm* G12C, such as sotorasib or adagrasib, which currently have a therapeutic impact in clinical practice for metastatic lung cancer (20, 21). Other therapies targeting the *KRASm* G12C or other *KRAS* mutations, as single agents or in combination, are being investigated in clinical trials (22).

Because of the hypothetical radioresistance of *KRASm* that may reduce the efficacy of CRT and the existence of targeted *KRASm* G12C therapies that may counteract these resistance mechanisms, our study aimed to evaluate the impact of *KRASm* on the efficacy of CRT in patients with unresectable stage II or III NSCLC.

## Methods

### Objectives

The primary objective was to evaluate the response rate to CRT in patients with unresectable stage II or III NSCLC according to *KRASm* G12C status. The primary endpoint was the objective response rate (ORR), assessed using the number of complete response (CR) or partial response (PR) compared to the total number of patients for *KRASm* G12C NSCLC compared to *KRAS* wild-type (*KRASwt*). The ORR was chosen as the best way to evaluate response to CRT, particularly in relation to the hypothetical radioresistance induced by KRASm. ORR at the first assessment was not affected by the addition of durvalumab, unlike survival data. The secondary objectives were to assess the differences in ORR and disease control rate (DCR) to chemoradiotherapy for *KRASm* compared to *KRAS* wild-type, *KRASm* G12C compared to *KRASm* non-G12C, and *KRASm* compared to *KRASwt*. Another aim was to evaluate the impact of *KRASm* on overall survival (OS), progression-free survival (PFS), time to local relapse (TTLR), and time to distant relapse (TTDR). Finally, the impact of main co-mutations, such as *KRAS/TP53* (*KP*) and *KRAS/STK11* (*KL*), on the response to CRT, as well as their impact on OS and PFS, was evaluated.

### Patient selection

A retrospective observational multicenter study of unresectable stage II or III NSCLC treated with CRT was conducted in four French hospitals between January 2014 and December 2022. The inclusion criteria were age ≥ 18 years, patients with unresectable stage II or III NSCLC and treated with concomitant or sequential CRT, and patients who did not object to the use of their medical data for cancer research purposes. The exclusion criteria were the absence of *KRASm* status (molecular biology not performed or not available), patient refusal to participate, the absence of chemotherapy, and total radiotherapy dose <60 Gy isoeffective in 2-Gy fractions (EQD2), as this threshold corresponds to a curative dose in the included patients. Genomic testing for *KRASm* was performed using next-generation sequencing (NGS) or broad panels based on polymerase chain reaction. This study was conducted in accordance with the French Research Standard MR-004 “Research not involving human participants” and is registered with the French Health Data Hub under the reference F20211126103028.

### Data collection

Patient characteristics included the World Health Organization (WHO) performance status prior to CRT, smoking habit (never, current, and former), and demographic data such as age and sex. Former smokers were defined as those who ceased smoking over a period of at least 1 year. Lung cancer characteristics included histology, TNM stage, positron emission tomography (PET) scan imaging, history of lung cancer, history of lung radiotherapy, history of lung cancer surgery, and programmed death-ligand 1 (PD-L1) status expressed as a percentage of positive tumor cells: negative (<1%), low (1%–50%), and high (>50%). Collected radiotherapy modalities were treatment duration, total dose, dose per fraction, number of fractions, and technique (3D conformal versus intensity-modulated radiotherapy). Data on chemotherapy included concomitant or sequential chemotherapy, the type of chemotherapy, the presence of induction chemotherapy only for patients treated with a concomitant strategy, and the number of cycles of platinum-based chemotherapy (one cycle of platinum-based chemotherapy according to the following charts: D1 = D22 or D1, D8, D15, D1 = D22). The following were also collected: mutation status, presence of consolidation immunotherapy, date of relapse (regardless of local or metastatic relapse site), date of local relapse, date of metastatic relapse, date of death, and causes of death. Treatment response was assessed using CT scan reports of complete or partial response, and stable or relapsed disease, based on Response Evaluation Criteria in Solid Tumors (RECIST) 1.1. The response to treatment was evaluated based on the first CT scan performed after CRT.

### Statistical analysis

ORR was defined as the percentage of patients who achieved a response, which could be either a complete response or a partial response. DCR was defined as the percentage of patients who achieved a response or stability. PFS was defined from the start of chemotherapy until the date of relapse, date of death of any cause, or date of last news. OS was defined from the start of chemotherapy until death from any cause or last news. TTLR was defined as the time from the start of chemotherapy to local relapse, and TTDR was defined as the time from the start of chemotherapy to distant relapse.

Descriptive statistics were mean, standard deviation, median, extreme values for continuous data, and frequencies and percentages for categorical data.

OS, PFS, TTLR, and TTDR were estimated using the Kaplan–Meier method, and comparisons were made using the log-rank test. Proportional Cox regression was used to evaluate the association between time-to-event outcomes and covariates.

All tests were two-sided; p-values <0.05 were considered statistically significant. All analyses were performed in R version 4.1.2.

## Results

### Description of the study population

**Figure 1:**
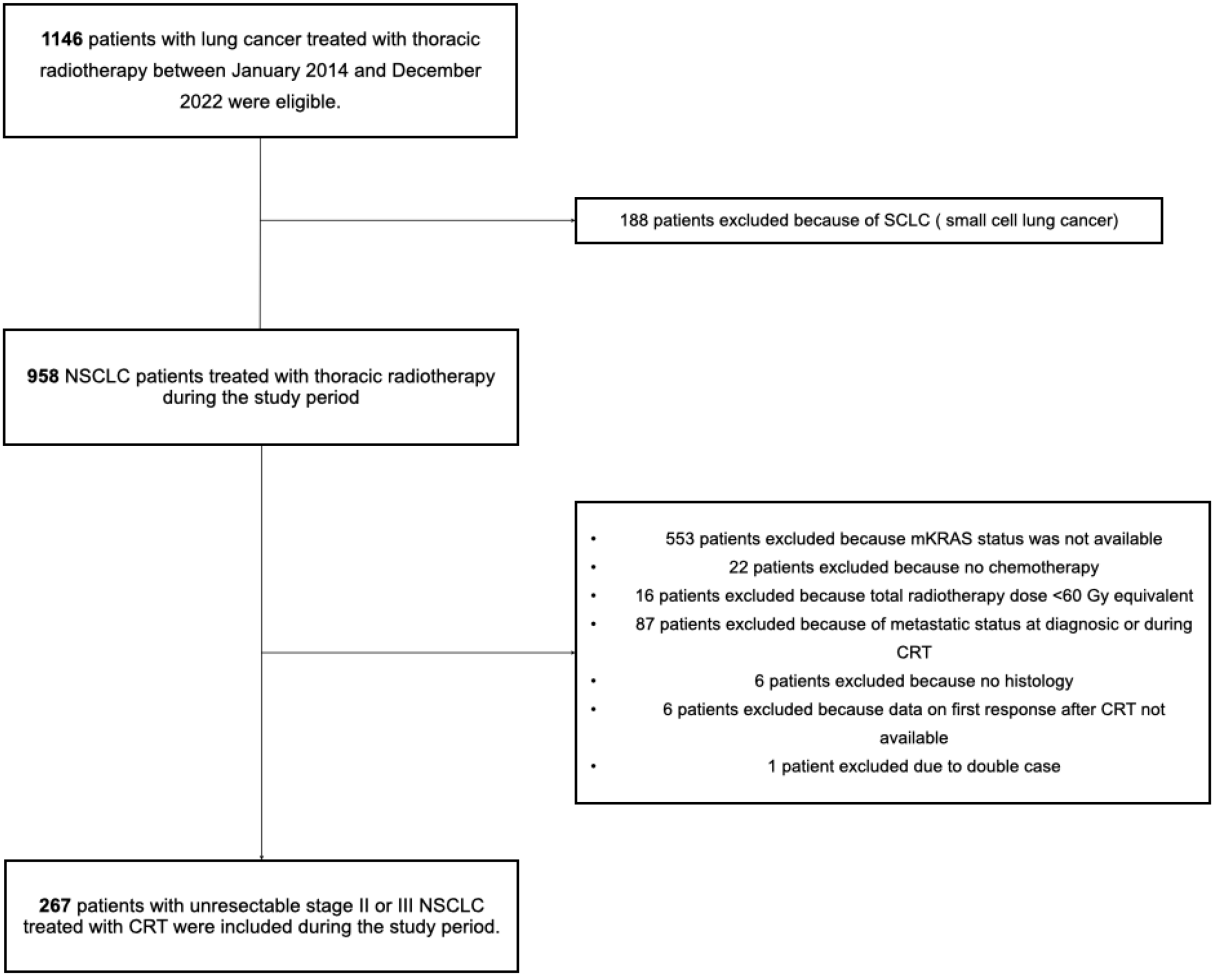
Study flowchart. SCLC, small cell lung cancer; NSCLC, non-small cell lung cancer; *KRASm*, KRAS mutation; CRT, chemoradiotherapy.

A total of 267 patients with unresectable stage II or III NSCLC treated with CRT were included in our study (**Figure 1**), of which 73 had *KRASm* (27.3%). The three most common *KRASm*s were *KRAS* G12C (n = 42, 15.7%), *KRAS* G12V (n = 13, 4.9%), and *KRAS* G12D (n = 7, 2.6%). In the *KRASm* population, a co-mutation with *TP53* and *STK11* in cases where the mutation status was known was reported in 45.5% (n = 20/44) and 7% (n = 4/57), respectively. For molecular alterations for which molecular status was available, excluding *KRASm*, the most commonly reported molecular alterations (with a prevalence >5%) were *TP53* in 58.1% (n = 97/167), *BRAF* in 6.4% (n = 17/265), *STK11* in 6.4% (n = 14/219), and *EGFR* in 5.6% (n = 15/266) (**Supplementary Table 1**, **Supplementary Figure 1**). The median age was 63.8 years (range, 30.9–83.0), and there was a male predominance (n = 177, 66%). Performance status (PS) was 0 or 1 for 96% (n = 257) and a current or former smoking status for 94% (n = 244) of patients. Lung adenocarcinoma was observed in 91% (n = 244), and the remainder were not otherwise specified (NOS) NSCLC (n = 23, 9%). Eight percent (n = 21) of patients had stage II disease (n = 21) and 92% (n = 232) stage III, with a predominance of stage IIIA (n = 101, 40%) and stage IIIB (n = 104, 41%). Seventy-five percent (n = 200) of patients were treated with concomitant CRT, and induction chemotherapy was given in 90% (n = 180) of the patients. All but one patient received platinum-based concurrent chemotherapy, predominantly carboplatin only (n = 210, 78.5%), with a mean number of platinum cycles of 3.67 (range, 0–8 cycles). Taxane chemotherapy was reported in 54% (n = 144) and pemetrexed in 49% (n = 130) (**Supplementary Table 2**). The median duration of radiotherapy was 47 days (range, 32–92), and the median dose was 66 Gy (range, 55–86) with a median dose per fraction of 2 Gy (range, 2–2.75). PD-L1 status was negative, 1%–49%, >50%, and unknown in 40% (n = 74), 35% (n = 64), 25% (n = 45), and 31.5% (n = 84), respectively. The administration of durvalumab consolidation was reported in 28% (n = 76) (**Table 1** for general features of the population study).

**Table 1:** General features of the study population.

Patients characteristics	*KRAS*wt	*KRASm* non-G12C	*KRASm* G12C	Total	p
	(N = 194)	(N = 31)	(N = 42)	(N = 267)	
Age					0.964
Median (min–max)	64.1 (30.9–83.0)	63.0 (48.2–75.2)	62.6 (38.2–77.6)	63.8 (30.9–83.0)	
Sex (%)					0.15
Male/female	135 (70)/59 (30)	19 (61)/12 (39)	23 (55)/19 (45)	177 (66)/90 (34)	
PS (%)					0.487
0	45 (23)	7 (23)	12 (29)	64 (24)	
1	139 (72)	24 (77)	30 (71)	193 (72)	
2	10 (5)	0 (0)	0 (0)	10 (4)	
Smoking status (%)					0.206
Current	96 (51)	19 (63)	22 (54)	137 (53)	
Former	77 (41)	11 (37)	19 (46)	107 (41)	
Never	14 (7)	0 (0)	0 (0)	14 (5)	
Missing	7 (3.6)	1 (3.2)	1 (2.4)	9 (3.4)	
History of lung cancer surgery (%)					0.575
No/yes	179 (92)/15 (8)	28 (90)/3 (10)	37 (88)/5 (12)	244 (91)/23 (9)	
History of lung radiotherapy (%)					0.123
No/yes	193 (100)/0 (0)	29 (97)/1 (3)	41 (100)/0 (0)	263 (100)/1 (0)	
Missing	1 (0.5)	1 (3.2)	1 (2.4)	3 (1.1)	
PET (%)					0.081
No/yes	0 (0)/194 (100)	1 (3)/30 (97)	1 (2)/41 (98)	2 (1)/265 (99)	
Histology (%)					0.086
Adenocarcinoma/NOS	173 (89)/21 (11)	31 (100)/0 (0)	40 (95)/2 (5)	244 (91)/23 (9)	
Stage (%)					0.742
II	16 (9)	1 (4)	4 (10)	21 (8)	
IIIA	78 (42)	8 (31)	15 (38)	101 (40)	
IIIB	72 (39)	15 (58)	17 (42)	104 (41)	
IIIC	21 (11)	2 (8)	4 (10	27 (11)	
Missing	7 (3.6)	5 (16.1)	2 (4.8)	14 (5.2)	
RT technique (%)					0.018*
3D/IMRT	54 (28)/140 (72)	2 (6)/29 (94)	13 (31)/29 (69)	69 (26)/198 (74)	
RT duration, days					0.533
Median (min–max)	47.0 (32.0–93.0)	49.0 (43.0–63.0)	47.0 (38.0–58.0)	47.0 (32.0–93.0)	
RT dose, Gray					0.626
Mean (SD)	65.8 (2.10)	65.4 (2.08)	65.9 (1.49)	65.8 (2.10)	
Median (min–max)	66.0 (55.0–86.0)	66.0 (56.0–66.0)	66.0 (60.0–70.0)	66.0 (55.0–86.0)	
Type of CRT (%)					0.695
Sequential/concomitant	51 (26)/143 (74)	6 (19)/25 (81)	10 (24)/32 (76)	67 (25)/200 (75)	
N platinum salt					0.034*
Mean (min–max)	3.59 (0–8.00)	3.65 (2.00–7.00)	4.10 (2.00–8.00)	3.67 (0–8.00)	
PD-L1 status (%)					0.573
Negative (<1%)	56 (41)	7 (30)	11 (44)	74 (40)	
Low (1%–50%)	49 (36)	9 (39)	6 (24)	64 (35)	
High (>50%)	30 (22)	7 (30)	8 (32)	45 (25)	
Missing	59 (30.4)	8 (25.8)	17 (40.5)	84 (31.5)	
Durvalumab (%)					0.167
Yes	50 (26)	13 (42)	13 (31)	76 (28)	
ORR (%)					0.895
No/yes	98 (51)/96 (49)	17 (55)/14 (45)	22 (52)/20 (48)	137 (51)/130 (49)	
DCR (%)					0.664
No/yes	162 (84)/32 (16)	28 (90)/3 (10)	36 (86)/6 (14)	226 (85)/41 (15)	

p-Value considered statistically significant was less than 0.05.

*KRASwt*, wild-type KRAS; KRASm non-G12C, *KRAS* mutations excluding G12C; KRASm G12C, *KRAS* G12C mutation; SD, standard deviation; CRT, chemoradiotherapy; RT, radiotherapy; PD-L1, programmed death-ligand 1; N, number; NOS, not otherwise specified; PS, performance status; PET, positron emission tomography; WHO status, World Health Organization status; IMRT, intensity-modulated radiotherapy; ORR, objective response rate; DCR, disease control rate; p-value considered statistically significant was less than 0.05. The values in bold are statistically significant.

### *KRASm* and therapeutic response to chemoradiotherapy (*KRASm* G12C, *KRASm* non-G12C, and *KRASm*)

There was no difference between *KRASm* G12C and *KRASwt* patients in terms of ORR (48% vs. 49%; p = 0.961) and DCR (86% vs. 84%; p = 0.903) (**Supplementary Table 3**).

There was no difference between *KRASm* and *KRASwt* patients in terms of ORR (47% vs. 49%; p = 0.78) and DCR (88% vs. 84%; p = 0.515) (**Supplementary Table 4**).

There was no difference between KRASm G12C and *KRASm* non-G12C in terms of ORR (48% vs. 45%); p = 1) and DCR (86% vs. 90%; p = 0.724) (**Supplementary Table 5**).

No difference was observed in the ORR or DCR between *KRASwt*, *KRASm* G12C, and *KRASm* non-G12C (p = 0.895 for ORR and p = 0.664 for DCR) (**Table 1**).

### *KRASm* and survival

**Figure 2:**
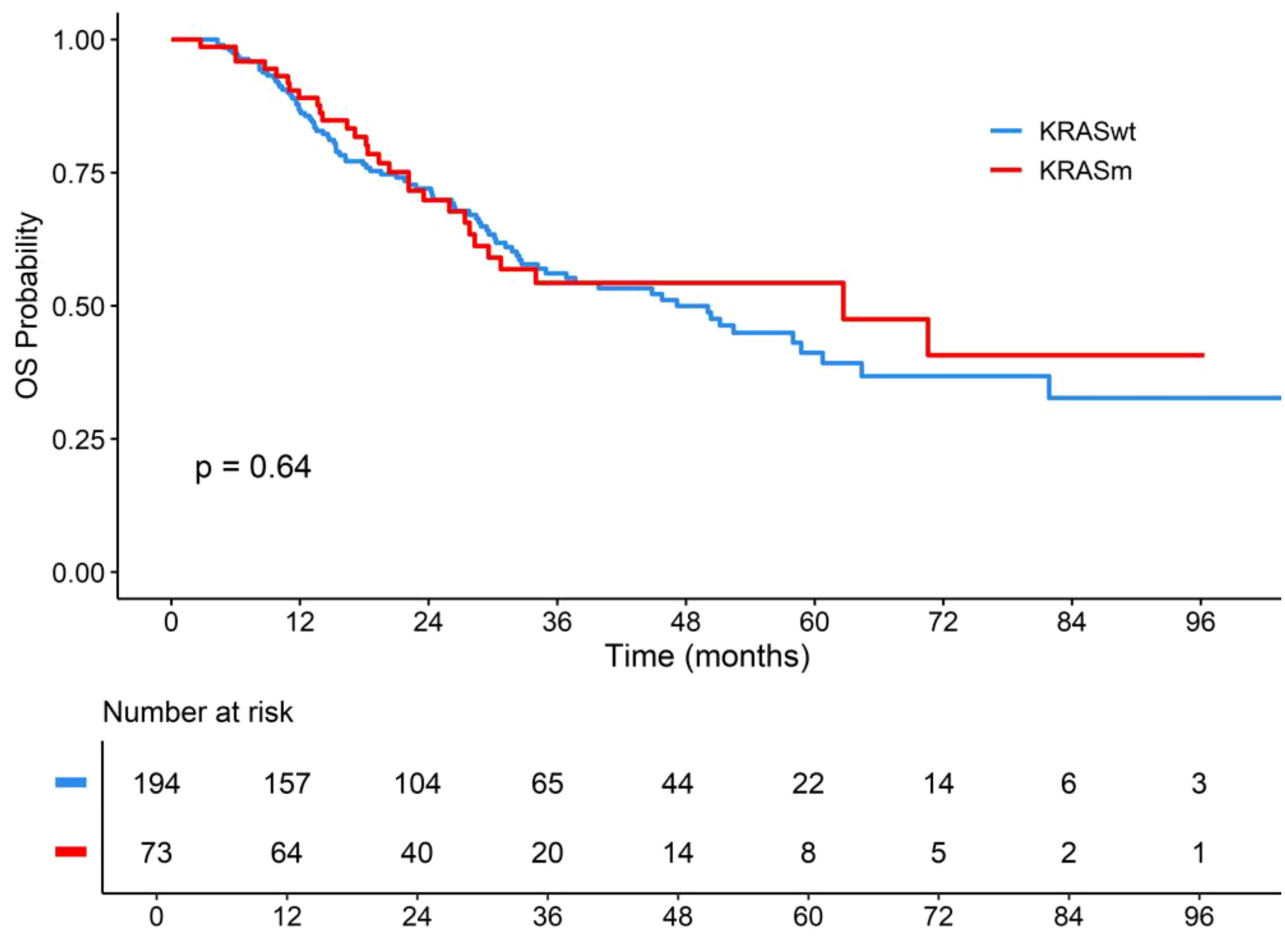
OS according to *KRAS* mutation status in NSCLC treated with chemoradiotherapy. OS, overall survival; KRASwt, KRAS wild-type; KRASm, KRAS mutation; NSCLC, non-small cell lung cancer. p-Value considered statistically significant was less than 0.05.

**Figure 3:**
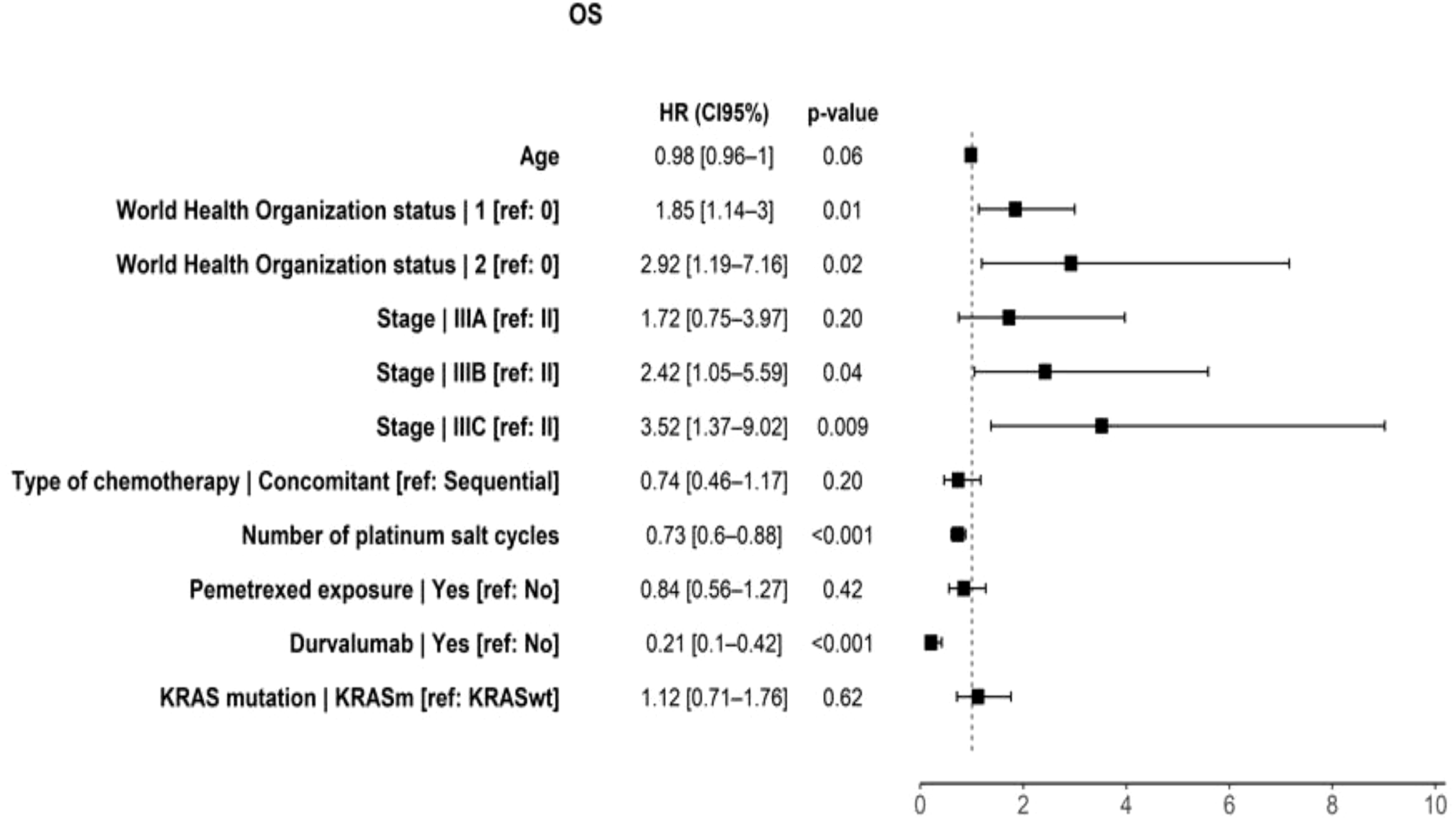
Prognostic factors associated with OS in multivariate analysis. OS, overall survival; HR, hazard ratio; *KRASm*, KRAS mutation; *KRASwt*, KRAS wild-type. p-Value considered statistically significant was less than 0.05.

There was no difference in OS between *KRASm* and *KRASwt* (p = 0.64). However, there was a numerical benefit for *KRASm* with a median OS (mOS) of 62.7 months for *KRASm* (95% CI, 28.3–NA) and an estimated 5-year survival rate of 54.3%. In contrast, the mOS was 47.2 months (95% CI, 34.2–64.4), and the estimated 5-year survival rate was 41.2% for *KRASwt* patients (**Figure 2**). On multivariate analysis, there was no association between *KRASm* and OS [hazard ratio (HR) = 1.13, p = 0.61] (**Figure 3**).

**Figure 4:**
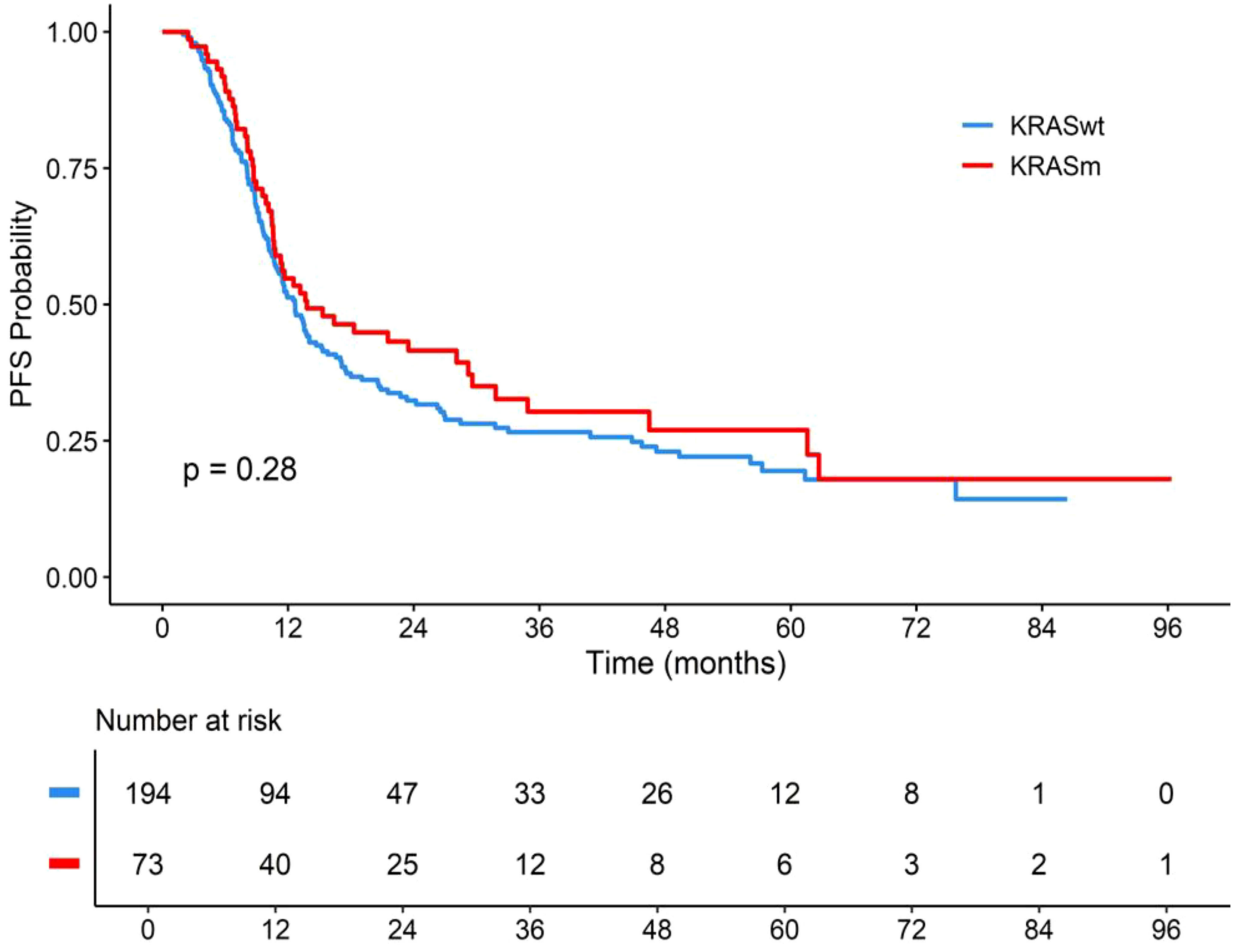
PFS according to *KRAS* mutation status in NSCLC treated with chemoradiotherapy. PFS, progression-free survival; KRASwt, KRAS wild-type; KRASm, KRAS mutation; NSCLC, non-small cell lung cancer. p-Value considered statistically significant was less than 0.05.

**Figure 5:**
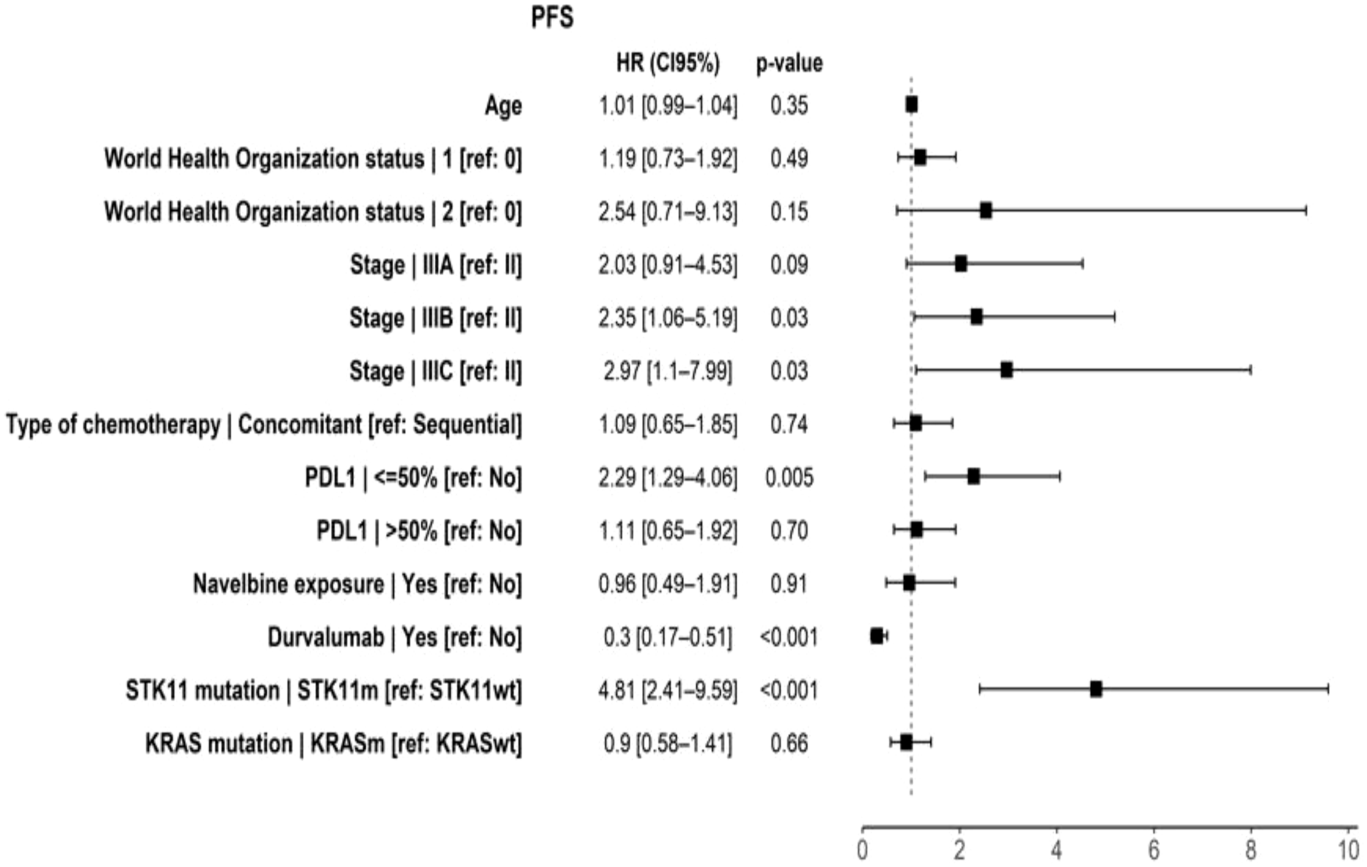
Prognostic factors associated with PFS in multivariate analysis. PFS, progression-free survival; WHO status, World Health Organization status; PD-L1, programmed death-ligand 1; *KRASm*, *KRAS* mutation; *STK11m*, *STK11* mutation; HR, hazard ratio. p-Value considered statistically significant was less than 0.05.

There was no difference in PFS between *KRASm* and *KRASwt* (p = 0.28), with median PFS of 13.8 months for *KRASm* (95% CI, 10.8–29.6) and 12.7 months for *KRASwt* (95% CI, 11.0–15.2), and estimated 5-year PFS of 27% for *KRASm* and 19.5% for *KRASwt* (**Figure 4**). On multivariate analysis, there was no association between *KRASm* and PFS (HR = 1.01, p > 0.9) (**Figure 5**).

No difference was observed in OS or PFS between *KRASwt*, *KRASm* G12C, and *KRASm* non-G12C (p = 0.89 for OS and p = 0.27 for PFS) (**Supplementary Figures 2**, **3**).

### *KRASm* and local/distant relapses

There was no difference in TTLR when comparing *KRASm* to *KRASwt* (p = 0.26), with a median TTLR of 26.9 months (95% CI, 18.3–40.3) for *KRASm* and 18.2 months for *KRASwt* (95% CI, 15.2–26.5) (**Supplementary Figure 4**). There was no difference in TTDR (p = 0.3) with a median of 25.9 months (95% CI, 16.4–NA) for *KRASm* and 17.1 months for *KRASwt* (95% CI, 13.8–26.5) (**Supplementary Figure 5**).

On multivariate analysis, there was no association between *KRASm* and TTLR (HR = 0.86, p = 0.4) or TTDR (HR = 0.86, p = 0.44) (**Supplementary Figure 6** for TTLR and **Supplementary Figure 7** for TTDR).

### Results for durvalumab (subgroup analysis and all cases)

On multivariate analysis, durvalumab was associated with better OS (HR = 0.21, p < 0.001), PFS (HR = 0.3, p < 0.001), TTLR (HR = 0.39, p < 0.001), and TTDR (HR = 0.32, p < 0.001) (**Figures 3**, **5**; **Supplementary Figures 6**, **7**).

There was no difference in OS, PFS, TTLR, and TTDR after adjusting for the presence of durvalumab when comparing *KRASm* to *KRASwt* (OS, p = 0.44; PFS, p = 0.97; TTLR, p =0.65; TTDR, p = 0.86) (**Supplementary Figures 8a**, **9a**, **10a**, **11a**).

For all patients treated with durvalumab, durvalumab was associated with better OS (p < 0.0001) and PFS (p < 0.0001) compared to patients not treated with durvalumab, with estimated 5-year rates of 75.3% and 37.7% for OS and PFS, respectively (**Supplementary Figures 12**, **13**).

### The impact of KP and KL on therapeutic response and survival outcomes (OS and PFS)

There were no differences observed between *KP* and *KP* wild-type (*KPwt*) in terms of ORR (45% vs. 50%; p = 0.92), but there was a significant trend in favor of the *KP* group regarding DCR (100% vs. 85%; p = 0.09) (**Supplementary Figure 14**). There was also no significant difference in OS and PFS, although there was a trend toward better PFS in favor of the *KP* group (p = 0.64 for OS and 0.12 for PFS) (**Supplementary Figures 15**, **16** for OS and PFS, respectively).

There were no differences in therapeutic response or OS between *KL* and *KL* wild-type (*KLwt*) (ORR, p = 0.62; DCR, p = 1; OS, p = 0.84). However, PFS was worse in the KL group (p = 0.029) (**Supplementary Figures 17**–**19** for therapeutic response, OS, and PFS, respectively).

### Prognostic factors associated with OS and PFS

The results of the univariate analysis are available in **Supplementary Figure 20**.

As previously mentioned, on multivariate analysis, the presence of durvalumab in consolidation was associated with improved OS (HR = 0.21, p < 0.001) and a greater number of platinum salt cycles (HR = 0.73, p < 0.001). A poorer OS was observed in patients with more advanced disease (stage IIIB or IIIC) (IIIB, p = 0.04; IIIC, p = 0.009) and an impaired general condition (PS 1 or 2 vs. PS 0) (PS 1, p = 0.01; PS 2, p = 0.02).

The only factor associated with improved PFS was the presence of durvalumab (HR = 0.3, p < 0.001). The following factors were associated with poorer PFS: the presence of an STK11 mutation (HR = 4.81, p < 0.001), low PD-L1 status (HR = 2.29, p = 0.005), and advanced-stage IIIB/IIIC disease (IIIB, p = 0.03; IIIC, p =0.03).

## Discussion

In our cohort of 267 patients with unresectable stage II or III NSCLC treated with CRT, approximately 27% had *KRASm* (n = 73), with a predominance of the *KRASm* G12C subtype (57.5% of all *KRASm*). These frequencies are consistent with those reported previously in large-scale molecular profiling studies of NSCLC (7, 8).No significant differences were observed between KRASm and KRASwt patients with respect to ORR (p = 0.78) or DCR (p = 0.515). Similarly, no differences were observed in terms of OS (p = 0.64), PFS (p = 0.28), TTLR (p = 0.26), and TTDR (p = 0.3) between KRASm and KRASwt. The prognostic impact of KRASm in NSCLC is still being debated. A meta-analysis of 41 studies involving 6,939 patients with NSCLC reported an unfavorable effect of KRASm on survival (HR = 1.45; 95% CI, 1.58-2.44). Subgroup analysis by stage revealed that *KRASm* was associated with poorer prognosis in early-stage disease: stage I (1.81; 95% CI, 1.36–2.39) and stage I–IIIa (1.68; 95% CI, 1.11–2.55), but not in advanced-stage disease (IIIb–IV) (1.3; 95% CI, 0.99–1.71). These findings suggest that KRAS mutations increase the risk of recurrence after resection but do not significantly affect the response to chemoradiotherapy and systemic therapies in stages III/IV (23). A retrospective study of 119 patients, including 16 with *KRASm* (13%), reported lower response rates to CRT among patients with *KRASm* compared to those with *KRASwt* (63% vs. 81%) (19). Another retrospective study of unresectable stage III NSCLC treated with CRT, including 114 *KRASwt* patients and 42 *KRASm* patients with 48% *KRASm* G12C (n = 20), reported that *KRASm* patients were less likely to receive immune checkpoint inhibitor (ICI) consolidation due to rapid disease progression post-CRT (23.8% vs. 4.4%, p = 0.007). In the Barsouk study, *KRASm* patients had worse PFS (median 6.3 vs. 10.7 months, p = 0.041) but similar OS (median 23.1 vs. 27.3 months, p = 0.237) (24).

However, the baseline characteristics of the studies varied. The Yagishita study included only Asian patients and had a male predominance (n = 12, 75%), whereas our cohort was balanced (58% of male patients), and the Barsouk study reported a predominance of female patients (n = 29, 69%).

In contrast, our study of a large cohort did not show any negative impact of *KRASm* on CRT response. Notably, prior studies did not specifically evaluate the impact of *KRASm* G12C on therapeutic outcomes. To our knowledge, our analysis is one of the few that directly evaluates this association. There were no significant differences in ORR (p = 0.961) or DCR (p = 0.903) between *KRASm* G12C and *KRASwt* patients. Likewise, OS and PFS did not differ among *KRASwt*, *KRASm* G12C, and *KRASm* non-G12C patients (p = 0.89 for OS and p = 0.27 for PFS). A recent systematic review and meta-analysis in NSCLC, *KRASm* G12C tumors had worse OS (HR = 1.42; 95% CI, 1.10–1.84, p = 0.007) but similar DFS (HR = 2.36, 95% CI 0.64–8.16) compared to *KRASwt* tumors. When compared to other *KRAS* mutations, *KRASm* G12C tumors had worse DFS (HR = 1.49; 95% CI, 1.07–2.09, p < 0.0001) but similar OS (HR = 1.03; 95% CI, 0.84–1.26). However, substantial heterogeneity and potential publication bias limit the robustness of these findings (11). Sebastian et al. analyzed a large real-world German cohort of 1,039 patients, including 160 patients with *KRASm* G12C (15.4%) and 251 patients with *KRASm* non-G12C (24.2%). They found no significant differences in clinical outcome between *KRASwt*, G12C, and non-G12C mutations; *KRAS* mutation status was not prognostic in the model. However, only a small proportion of NSCLC had non-metastatic disease (eight for *KRASm* G12C, 14 for *KRASm* non-G12C, and 43 for *KRASwt*). These findings are consistent with those of several other studies (25–27).

We did not select either OS or PFS as the primary endpoint because the results would have been biased by the introduction of consolidation immunotherapy in 2017. In the PACIFIC study, durvalumab immunotherapy induced significant improvements in OS and PFS compared to placebo. The estimated 5-year OS and PFS rates were 42.9% and 33.1%, respectively, in the durvalumab arm (2). In our real-world study, durvalumab was associated with improved OS (HR = 0.21, p < 0.001) and PFS (HR = 0.3, p < 0.001). We observed a 5-year OS of 75.3% and a 5-year PFS of 37.7%. The comparable 5-year PFS and improved 5-year OS were attributed to the smaller sample size. After adjusting for durvalumab, we found no difference in OS (p = 0.44), PFS (p = 0.97), TTLR (p = 0.65), or TTDR (p = 0.86) between KRASm and KRASwt patients, with 26 KRASm patients receiving ICI consolidation therapy. Similar results were reported in the Barsouk study, which included 29 KRASm patients (PFS, 8.1 vs. 11.9 months, p = 0.35; OS, 30.5 vs. 31.7 months, p = 0.692), as well as in the Guo study, which included 18 KRASm patients (PFS, 12.6 vs. 12.7 months, p = 0.77; OS, 20.0 vs. 32.4 months, p = 0.69). However, divergent results were reported in the Liu study, which included 22 KRASm patients. These patients had worse PFS (8 vs. 40.1 months, p < 0.001) but similar OS (36.2 months vs. NA, p = 0.07) in the KRAS subgroup (24, 28, 29).

We will analyze the impact of the main co-mutations (*KP* and *KL*) in parallel. No differences were found in terms of DCR or ORR between *KP* and *KPwt*, nor between *KL* and *KLwt* (p = 0.09 and p = 1, respectively, for DCR; p = 0.92 and p = 0.84, respectively, for ORR). There were no differences in OS between *KP* and *KPwt* (p = 0.64), nor between KL and *KLwt* (p = 0.84). There was a trend toward better PFS in favor of the *KP* group (p = 0.12) and worse PFS in the *KL* group (p = 0.029). Additionally, we know that co-occurring genetic events can impact the immune environment. *KP* was associated with the increased expression of PD-L1 and mutational burden, showing a remarkable clinical benefit for PD-1 inhibitors (30), while *KL* was significantly associated with PD-L1 negative in tumor mutational burden intermediate-high and represents a major driver of primary resistance to PD-1 blockade in *KRAS* mutant (8). *STK11* mutation was associated with a poorer prognosis (31), a finding that was also observed in our study. In the multivariate analysis, the *STK11* mutation was associated with poorer PFS (HR = 4.81, p < 0.001). Poorer PFS was also found in the *KL* group, which may be explained by the lesser benefits from consolidation immunotherapy compared to *KP* switching. However, these data should be interpreted with caution, given the limited sample size of 20 *KP* patients and four *KL* patients in our study. Future studies with larger sample sizes are needed to confirm these findings.

Two *KRASm* G12C-targeting treatments have demonstrated efficacy and are currently in clinical use. The first, sotorasib, is a *KRAS* G12C inhibitor that significantly increased PFS and had a more favorable safety profile than docetaxel in patients with advanced NSCLC harboring the KRASm G12C mutation who had previously been treated with other anticancer drugs (20). The second is adagrasib, another *KRAS* G12C inhibitor. In a phase 2 study, it showed clinical activity with an objective response rate of 42.9%, a median duration of response of 8.5 months, and a median PFS of 6.5 months (21). Additionally, *KRASm* G12C tumors have been reported to exhibit higher PD-L1 expression than *KRASwt* tumors and may benefit from anti-PD-1/PD-L1 blockade (7, 32–34). Several studies have reported promising results from combining a *KRAS* G12C inhibitor with an ICI (35, 36). Recently, the primary analysis of the KRYSTAL-12 trial, with a median follow-up of 9.4 months, showed a significant improvement in PFS with adagrasib versus docetaxel (HR = 0.58; 95% CI, 0.45–0.76; p < 0.0001; median PFS 5.49 vs. 3.84 months) in patients with *KRASm* G12C locally advanced or metastatic NSCLC who had previously received platinum-based chemotherapy, concurrently or sequentially with anti-PD-(L)1 therapy (37).

A recent phase III study showed that osimertinib treatment significantly increased progression-free survival (median PFS, 39.1 months for osimertinib vs. 5.6 months for placebo) in patients with unresectable stage III EGFR-mutated NSCLC who did not experience progression during or after chemoradiotherapy (38). A similar approach could be promising for a therapy targeting *KRASm* G12C in combination with immunotherapy. Several earlier-stage studies are currently evaluating a neoadjuvant strategy that combines a *KRAS* G12C inhibitor with either chemotherapy or immunotherapy (NCT05118854 and NCT05118854).

The present study has several limitations that are specific to retrospective studies. These limitations include the national scope of the study, the small size of the cohorts, and the real-world nature of the available molecular data, which varies between centers. Our study’s data do not suggest that *KRASm* G12C or *KRASm* is radioresistant. There is no significant difference in ORR compared to *KRASwt*. However, the results of our study may be influenced by limited statistical power resulting from the small patient sample size, and there may be other associated factors, such as co-mutations, that affect the response to CRT. PD-L1 status was unavailable for 31.5% of the patients in our study (n = 84), which can be explained by the fact that the therapeutic impact of PD-L1 was initially limited to patients with PD-L1 ≥ 1%. Furthermore, the impact of ICI consolidation on survival outcomes according to KRAS status was limited, as only 25 KRASm patients and 13 KRASm G12C patients were treated with durvalumab. All these data must be compared with prospective data from larger samples.

## Conclusion

*KRAS* G12C mutation compared to KRAS wild-type did not affect response to chemoradiotherapy, and *KRAS* mutations compared to KRAS wild-type were not associated with worse survival in unresectable stage II or III NSCLC treated with chemoradiotherapy. Further studies are needed to investigate if co-mutations such as *KP* or *KL* could impact the response to chemoradiotherapy and survival in NSCLC.

## Data Availability

The original contributions presented in the study are included in the article/**Supplementary Material**. Further inquiries can be directed to the corresponding author.

## Supplementary material

Supplementary Table 1 Mutation status prevalence *KRASm*, *KRAS* mutation; KP, *KRAS*/*TP53* co-mutation; *KL*, *KRAS*/*STK11* co-mutation; *ALK*, anaplastic lymphoma kinase; *BRAF*, v-Raf murine sarcoma viral oncogene homolog B; *EGFR*, epithelial growth factor receptor; *HER2*, human epidermal growth factor receptor 2; *STK11*, serine/threonine kinase 11; *MET*, mesenchymal epithelial transition; *ROS-1*, c-ROS oncogene 1; *RET*, rearranged during transfection; *TP53*, tumor protein 53; *PIK3CA*, phosphatidylinositol-4,5-bisphosphate 3-kinase catalytic; there is a lack of data for certain genomic alterations due to heterogeneous molecular screening across centers for certain molecular alterations.

Supplementary Table 2 concomitant chemotherapy with platinum salts and type of platinum salts. *KRASm*, mutated *KRAS*; *KRASwt*, *KRAS* wild-type; ICI, immune check-point inhibitor; VEGF, vascular endothelial growth factor; EGFR, epithelial Growth Factor Receptor; CRT, chemoradiotherapy; some patients may have been treated as part of a clinical trial.

Supplementary Table 3 Comparison of *KRASm* G12C and *KRASwt* for response to chemoradiotherapy. *KRASm* G12c, *KRAS* G12C mutation; non-*KRASm* G12C, patients without *KRAS* G12C mutation; ORR, objective response rate; DCR, disease control rate; p-value considered statistically significant was less than 0.05.

Supplementary Table 4 Comparison of *KRASm* and KRASwt for response to chemoradiotherapy. *KRASm*, *KRAS* mutation; *KRASwt*, wild-type KRAS; ORR, objective response rate; DCR, disease control rate; p-value considered statistically significant was less than 0.05.

Supplementary Table 5 Comparison of *KRASm* G12C and KRASm non-G12C for response to chemoradiotherapy. *KRASm* G12c, *KRAS* G12C mutation; KRASm non-G12C, *KRAS mutations* excluding G12C; ORR, objective response rate; DCR, disease control rate; p-value considered statistically significant was less than 0.05.

Supplementary Figure 1 Spectrum of *KRASm. KRASm*, *KRAS* mutation; other *KRASm* - *KRASm* other than those shown in figure. *KRAS* G12S, *KRAS* G12F and G13D were present in 1.4%.

Supplementary Figure 2 OS comparison between *KRASwt*, *KRASm* G12C, and *KRASm* non-G12C. *KRASm non-G12C, KRAS mutation, excluding KRAS G12C; KRASm G12C, KRAS* G12C mutation *KRASm* G12C; *KRASwt, KRAS* wild-type; OS, overall survival; p-value considered statistically significant was less than 0.05.

Supplementary Figure 3 PFS comparison between *KRASwt*, *KRASm* G12C, and *KRASm* non-G12C. *KRASm non-G12C= KRAS mutation, excluding KRAS G12C; KRASm G12C, KRAS* G12C mutation *KRASm* G12C; *KRASwt, KRAS* wild-type; PFS, progression-free survival; p-value considered statistically significant was less than 0.05.

Supplementary Figure 4 TTLR according to *KRAS* mutation status in NSCLC treated by chemoradiotherapy. KRASwt, KRAS wild-type; KRASm, KRAS mutation; TTLR, time to local relapse; p-value considered statistically significant was less than 0.05.

Supplementary Figure 5 TTDR according to *KRAS* mutation status in NSCLC treated by chemoradiotherapy. KRASwt, KRAS wild-type; KRASm, KRAS mutation; TTDR, time to distant relapse; p-value considered statistically significant was less than 0.05.

Supplementary Figure 6 Prognostic factors associated with TTLR in multivariate analysis. TTLR, time to local relapse; WHO status, World Health Organization status; N platine, number of platinum salt cycles; HR, hazard ratio; CT, chemoradiotherapy; p-value considered statistically significant was less than 0.05.

Supplementary Figure 7 Prognostic factors associated with TTDR in multivariate analysis. TTDR, time to distant relapse; WHO status, World Health Organization status; N platine, number of platinum salt cycles; HR, hazard ratio; IMRT, intensity-modulated radiation therapy; CT, chemoradiotherapy; *KRASm*, *KRAS* mutation; p-value considered statistically significant was less than 0.05.

Supplementary Figure 8 Subgroup analysis for OS according to *KRASm*: durvalumab **(a)** and without durvalumab **(b)**. **(a)**. OS, overall survival; KRASwt, KRAS wild-type; KRASm= KRAS mutation; p-value considered statistically significant was less than 0.05. **(b)** OS, overall survival; KRASwt, KRAS wild-type; KRASm= KRAS mutation; p-value considered statistically significant was less than 0.05.

Supplementary Figure 9 Subgroup analysis for PFS according to *KRASm*: durvalumab **(a)** and without durvalumab **(b)**. **(a)** PFS, progression-free survival; KRASwt, KRAS wild-type; KRASm= KRAS mutation; p-value considered statistically significant was less than 0.05. **(b)** PFS, progression-free survival KRASwt, KRAS wild-type; KRASm= KRAS mutation; p-value considered statistically significant was less than 0.05.

Supplementary Figure 10 Subgroup analysis for TTLR according to *KRASm*: durvalumab **(a)** and without durvalumab **(b)**. **(a)** KRASwt, KRAS wild-type; KRASm= KRAS mutation; TTLR, time to local relapse; p-value considered statistically significant was less than 0.05. **(b)** KRASwt, KRAS wild-type; KRASm, KRAS mutation; TTLR, time to local relapse; p-value considered statistically significant was less than 0.05.

Supplementary Figure 11 Subgroup analysis for TTDR according to *KRASm*: durvalumab **(a)** and without durvalumab **(b)**. **(a)** KRASwt, KRAS wild-type; KRASm= KRAS mutation; TTDR= time to distant relapse; p-value considered statistically significant was less than 0.05. **(b)** KRASwt, KRAS wild-type; KRASm, KRAS mutation; TTDR, time to distant relapse; p-value considered statistically significant was less than 0.05.

Supplementary Figure 12 Subgroup analysis for OS according to durvalumab treatment OS, overall survival; p-value considered statistically significant was less than 0.05.

Supplementary Figure 13 Subgroup analysis for PFS according to durvalumab treatment. PFS, progression-free survival; p-value considered statistically significant was less than 0.05.

Supplementary Table 14 Comparison of *KP* and *KPwt* for response to chemoradiotherapy. *KP, KRAS/TP53 comutation*; *KPwt*, Patients without *KRAS* or *TP53* mutations; ORR, objective response rate; DCR, disease control rate; p-value considered statistically significant was less than 0.05.

Supplementary Table 15 OS comparison between *KP and KPwt. KP, KRAS/TP53 comutation*; *KPwt*, Patients without *KRAS* or *TP53* mutations; *KPwt*, both wild-type; WT, wild-type; OS, overall survival; p-value considered statistically significant was less than 0.05.

Supplementary Table 16 PFS comparison between *KP and KPwt. KP, KRAS/TP53 comutation*; *KPwt*, Patients without *KRAS* or *TP53* mutations; *KPwt*, both wild-type; WT, wild-type; PFS, progression-free survival; p-value considered statistically significant was less than 0.05.

Supplementary Table 17 Comparison of *KL* and *KLwt* for response to chemoradiotherapy. *KL, KRAS/STK11 comutation*; *KLwt*, Patients without *KRAS* or *STK11* mutations.; ORR, objective response rate; DCR, disease control rate; p-value considered statistically significant was less than 0.05.

Supplementary Table 18 OS comparison between *KL and KLwt. KL, KRAS/STK11 comutation*; *KLwt*, Patients without *KRAS* or *STK11* mutations; *KLwt*, both wild-type; WT, wild-type; OS, overall survival; p-value considered statistically significant was less than 0.05.

Supplementary Table 19 PFS comparison between *KL and KLwt. KL, KRAS/STK11 comutation*; *KLwt*, Patients without *KRAS* or *STK11* mutations; *KLwt*, both wild-type; WT, wild-type; PFS, progression-free survival; p-value considered statistically significant was less than 0.05.

Supplementary Table 20 - results of the univariate analysis on OS, PFS, TTLR and TTDR. OS, overall survival; PFS= progression-free survival; TTLR, time to local relapse; TTDR, time to distant relapse; *KRAS*, kirsten rat sarcoma; OS, overall survival; PS, performance status; HR, hazard ratio; IMRT, intensity-modulated radiation therapy; CRT, chemoradiotherapy; PD-L1, programmed death-ligand 1; *EGFR*, epithelial growth factor receptor; *BRAF*, v-Raf murine sarcoma viral oncogene homolog B; *ALK*, anaplastic lymphoma kinase; *STK11*, serine/threonine kinase 11; *RET*, rearranged during transfection; *TP53*, tumor protein 53; p-value considered statistically significant was less than 0.05.

## Glossary

*ALK*: anaplastic lymphoma kinase
*BRAF*: v-Raf murine sarcoma viral oncogene homolog B
CR: complete response
CRT: chemoradiotherapy
DCR: disease control rate
*EGFR*: epithelial growth factor receptor
*HER2*: human epidermal growth factor receptor 2
HR: hazard ratio
ICI: immune checkpoint inhibitor
IMRT: intensity-modulated radiation therapy
*KL*: co-mutation *KRAS/STK11*
*KP*: co-mutation *KRAS/TP53*
*KRAS*: Kirsten rat sarcoma
*KRASm*: *KRAS* mutation
*KRASm* G12C: *KRAS* G12C mutation
*KRASm* non-G12C: *KRAS* mutations excluding *KRAS* G12C mutation
*KRASwt*: *KRAS* wild-type
*MET*: mesenchymal epithelial transition
NGS: next-generation sequencing
NOS: not otherwise specified
NSCLC: non-small cell lung cancer
ORR: objective response rate
OS: overall survival
PCR: polymerase chain reaction
PD-L1: programmed death-ligand 1
PET: positron emission tomography
PFS: progression-free survival
*PIK3CA*: phosphatidylinositol-4,5-bisphosphate 3-kinase catalytic
PR: partial response
*RET*: rearranged during transfection
*ROS-1*: c-ROS oncogene 1
*STK11*: serine/threonine kinase 11
*TP53*: tumor protein 53
TTDR: time to distant relapse
TTLR: time to local relapse
*VEGF*: vascular endothelial growth factor
