# Supplementary material for: Impact of KRAS G12C mutation on the efficacy of chemoradiotherapy in patients with unresectable stage II or III non-small cell lung cancer
Source: Front Oncol. 2025 Nov 27;15:1675825. doi: 10.3389/fonc.2025.1675825 (PMC12695551; doi:10.3389/fonc.2025.1675825)
Supplement: Supplementary Table 1 — Mutation status prevalence KRASm, KRAS mutation; KP, KRAS/TP53 co-mutation; KL, KRAS/STK11 co-mutation; ALK, anaplastic lymphoma kinase; BRAF, v-Raf murine sarcoma viral oncogene homolog B; EGFR, epithelial growth factor receptor; HER2, human epidermal growth factor receptor 2; STK11, serine/threonine kinase 11; MET, mesenchymal epithelial transition; ROS-1, c-ROS oncogene 1; RET, rearranged during transfection; TP53, tumor protein 53; PIK3CA, phosphatidylinositol-4,5-bisphosphate 3-kinase catalytic; there is a lack of data for certain genomic alterations due to heterogeneous molecular screening across centers for certain molecular alterations. [file DataSheet1.pdf]

Supplementary data:

Supplementary table 1 - Mutation status prevalence

Mutation	*KRASm*	*KRASwt*	Total
	(N=73)	(N=194)	(N=267)
KRAS G12C	42 (57.5%)	0	42 (15.7%)
KRAS G12V	13 (17.8%)	0	13 (4.9%)
KRAS G12D	7 (9.6%)	0	7 (2.6%)
TP53	20/44 (45.5%)	77/123 (62.6%)	97/167 (58.1%)
STK11	4/57 (7%)	10/162 (6.2%)	14/219 (6.4%)
BRAF	2/72 (2.8%)	15/193 (7.8%)	17/265 (6.4%)
MET	0/64	7/176 (4%)	7/240 (2.9%)
PIK3CA	2/58 (3.5%)	5/167 (3%)	7/225 (3.1%)
RET	0/65	2/172 (1.2%)	2/237 (0.8%)
ROS-1	0/71	1/192 (0.5%)	1/263 (0.4%)
HER2	0/71	1/191 (0.5%)	1/262 (0.4%)
EGFR	2/72 (2.8%)	13 (6.7%)	15/266 (5.6%)
ALK	1/72 (1.4%)	4 (2.1%)	5/266 (1.9%)

KRASm = *KRAS* mutation; KP = *KRAS*/*TP53* co-mutation; *KL* = *KRAS*/*STK11* co-mutation; *ALK* = anaplastic lymphoma kinase; *BRAF* = v-Raf murine sarcoma viral oncogene homolog B; *EGFR* = epithelial growth factor receptor; *HER2* = human epidermal growth factor receptor 2; *STK11* = serine/threonine kinase 11 ; *MET* = mesenchymal epithelial transition; *ROS-1* = c-ROS oncogene 1; *RET* = rearranged during transfection; *TP53* = tumor protein 53 ; *PIK3CA* = phosphatidylinositol-4,5-bisphosphate 3-kinase catalytic; there is a lack of data for certain genomic alterations due to heterogeneous molecular screening across centers for certain molecular alterations

Supplementary table 2 - concomitant chemotherapy with platinum salts and type of platinum salts

	KRASm G12C (n=42)	*KRASm* (n=73)	KRASwt (n=194)	Total (n=267)
Platinium salts (%)				
Cisplatin only	6 (14.3)	10 (13.7)	26 (13.4)	36 (13.5)
Carboplatin only	33 (78.6)	56 (76.7)	154 (79.4)	**210 (78.7)**
Both Cisplatin and Carboplatin	3 (7.1)	7 (9.6)	13 (6.7)	20 (7.5)
No	0	0	1 (0.5)	1 (0.4)
Chemotherapy induction for concomitant CRT (%)				
Yes	31 (96.9)	51 (89.5)	129 (90.2)	**180 (90)**
No	1 (3.1)	6 (10.5)	14 (9.8)	20 (10)
Chemotherapy (%)				
Vinorelbine	10 (23.8)	15 (20.6)	26 (13.4)	41 (15.4)
Taxane	20 (47.6)	37 (50.7)	107 (55.2)	144 (53.9)
Pemetrexed	23 (54.8)	38 (52.1)	92 (47.4)	130 (48.7)
Gemcitabine	0 (0)	0 (0)	1 (0.5)	1 (0.4)
ICI (%)				
Pembrolizumab	1 (2.4)	2 (2.7)	3 (1.5)	5 (1.9)
Atezolizumab	0 (0)	0 (0)	1 (0.5)	1 (0.4)
Anti-VEGF therapy (%)				
Bevacizumab	0 (0)	0 (0)	1 (0.5)	1 (0.4)
Anti-EGFR (%)				
Cetuximab	0 (0)	0 (0)	1 (0.5)	1 (0.4)

KRASm – mutated *KRAS*; *KRASwt*= *KRAS* wild-type; ICI= immune check-point inhibitor; VEGF = vascular endothelial growth factor; EGFR = epithelial Growth Factor Receptor; CRT = chemoradiotherapy; some patients may have been treated as part of a clinical trial.

Supp.Table 3 - Comparison of *KRASm* G12C and *KRASwt* for response to chemoradiotherapy

	KRASwt	*KRASm* G12C	Total	p.
	(N=194)	(N=42)	(N=267)	
ORR				0.961
No	98(51%)	22 (52%)	137 (51%)	
Yes	96 (49%)	20 (48%)	130 (49%)	
DCR				0.903
No	32 (16%)	6 (14%)	41 (15%)	
Yes	162 (84%)	36 (86%)	226 (85%)	

KRASm G12c = *KRAS* G12C mutation; non-*KRASm* G12C = patients without *KRAS* G12C mutation; ORR = objective response rate; DCR = disease control rate; p-value considered statistically significant was less than 0.05.

Supp.Table 4 – Comparison of *KRASm* and KRASwt for response to chemoradiotherapy

	KRASwt	*KRASm*	Total	p.
	(N=194)	(N=73)	(N=267)	
ORR				0.774
No	98 (51%)	39 (53%)	137 (51%)	
Yes	96 (49%)	34 (47%)	130 (49%)	
DCR				0.515
No	32 (16%)	9 (12%)	41 (15%)	
Yes	162 (84%)	64 (88%)	226 (85%)	

KRASm = *KRAS* mutation; *KRASwt* = wild-type KRAS; ORR = objective response rate; DCR = disease control rate; p-value considered statistically significant was less than 0.05.

Supp.Table 5 - Comparison of *KRASm* G12C and KRASm non-G12C for response to chemoradiotherapy

	KRASm non-G12C	*KRASm* G12C	Total	p.
	(N=31)	(N=42)	(N=73)	
ORR				1
No	17 (55%)	22 (52%)	39 (53%)	
Yes	14 (45%)	20 (48%)	34 (47%)	
DCR				0.724
No	3 (10%)	6 (14%)	9 (12%)	
Yes	28 (90 %)	36 (86%)	64 (88%)	

KRASm G12c = *KRAS* G12C mutation; KRASm non-G12C = *KRAS mutations* excluding G12C; ORR = objective response rate; DCR = disease control rate; p-value considered statistically significant was less than 0.05.

Supplementary Figure 1 – Spectrum of *KRASm*

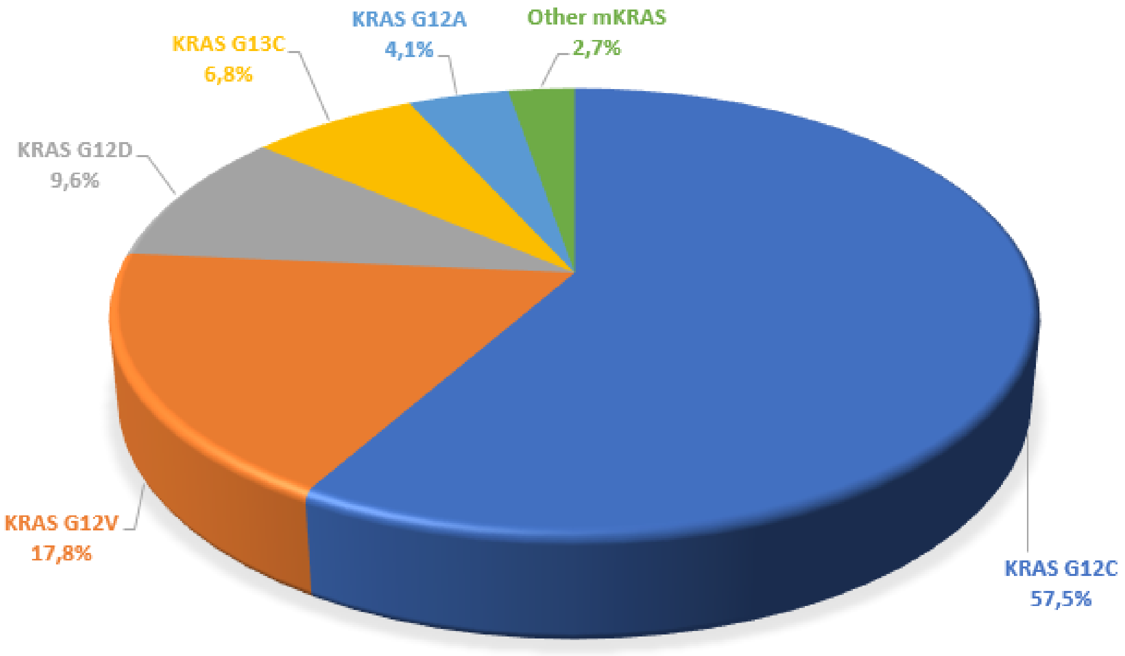

KRASm = *KRAS* mutation; other *KRASm* - *KRASm* other than those shown in figure

KRAS G12S, *KRAS* G12F and G13D were present in 1.4%.

Supplementary Figure 2 – OS comparison between *KRASwt*, *KRASm* G12C, and *KRASm* non-G12C

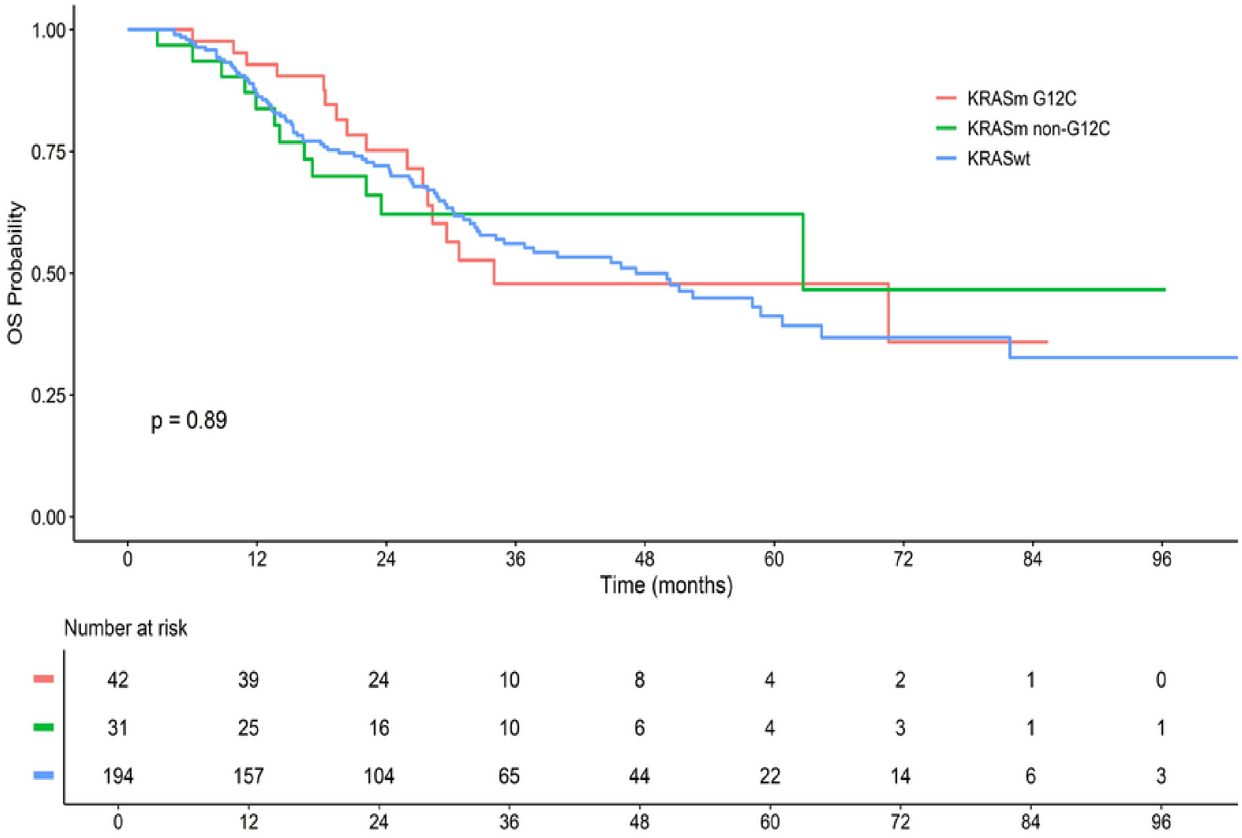

KRASm non-G12C= KRAS mutation, excluding KRAS G12C; KRASm G12C = KRAS G12C mutation *KRASm* G12C; *KRASwt* = *KRAS* wild-type; OS = overall survival; p-value considered statistically significant was less than 0.05.

.

Supplementary Figure 3 – PFS comparison between *KRASwt*, *KRASm* G12C, and *KRASm* non-G12C

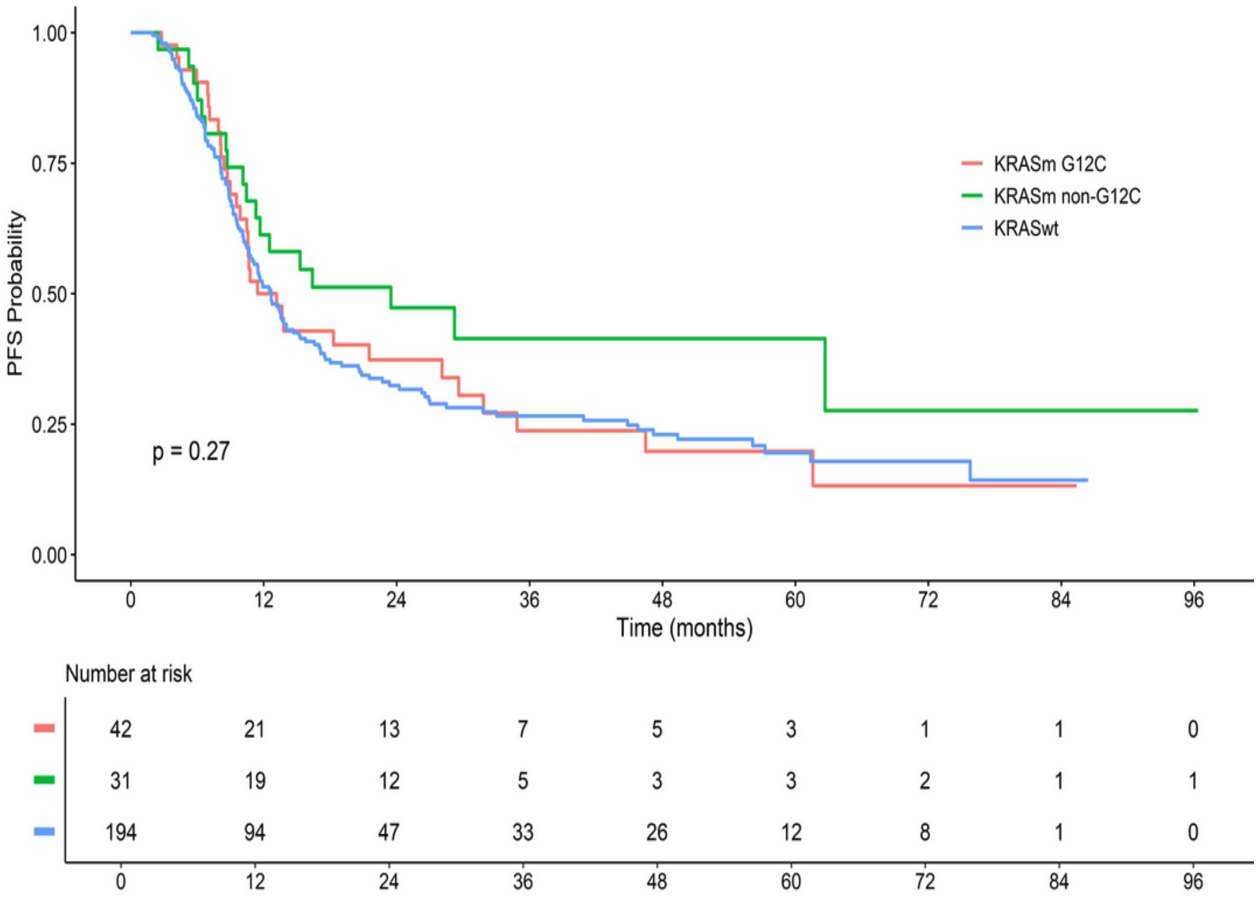

KRASm non-G12C= KRAS mutation, excluding KRAS G12C; KRASm G12C = KRAS G12C mutation *KRASm* G12C; *KRASwt* = *KRAS* wild-type; PFS = progression-free survival; p-value considered statistically significant was less than 0.05.

.

Supplementary Figure 4 – TTLR according to *KRAS* mutation status in NSCLC treated by chemoradiotherapy

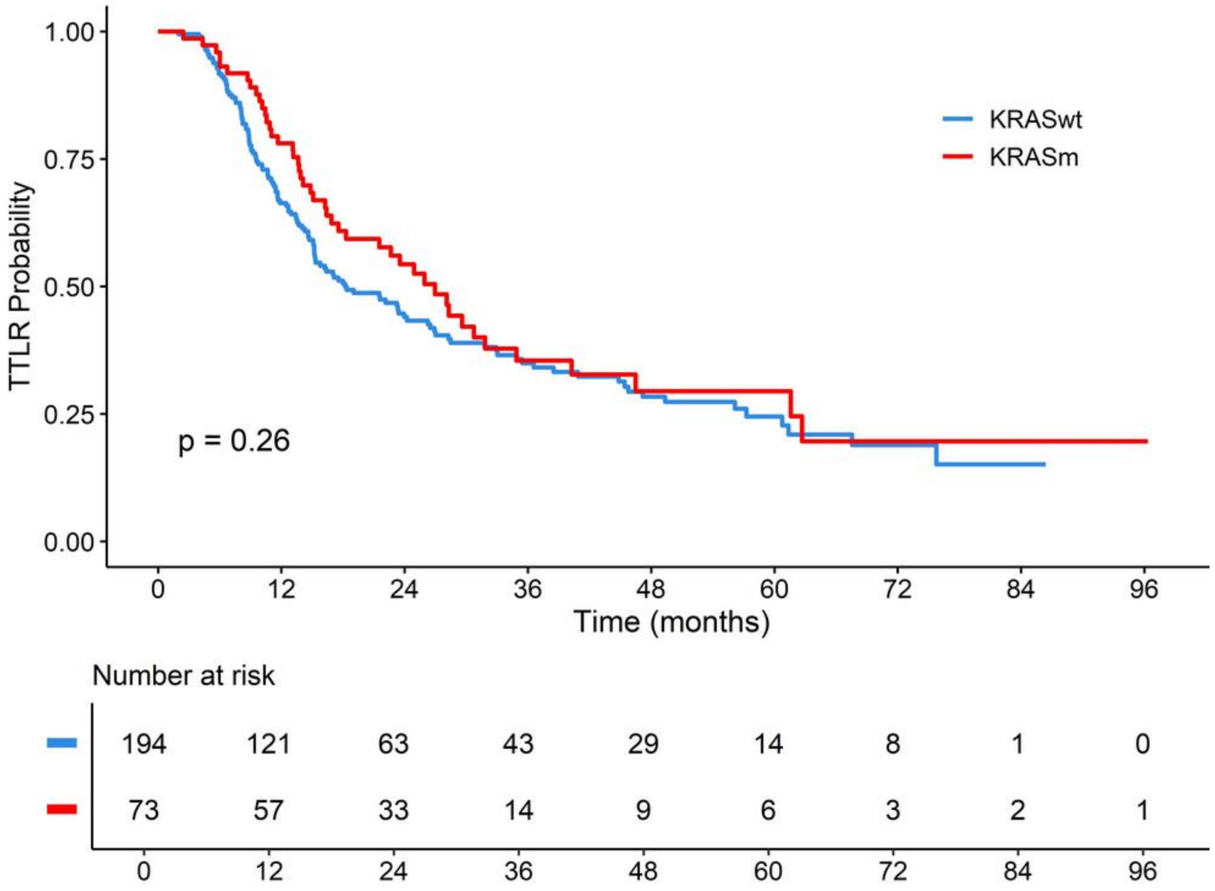

KRASwt = KRAS wild-type; KRASm= KRAS mutation; TTLR = time to local relapse; p-value considered statistically significant was less than 0.05.

Supplementary Figure 5 – TTDR according to *KRAS* mutation status in NSCLC treated by chemoradiotherapy

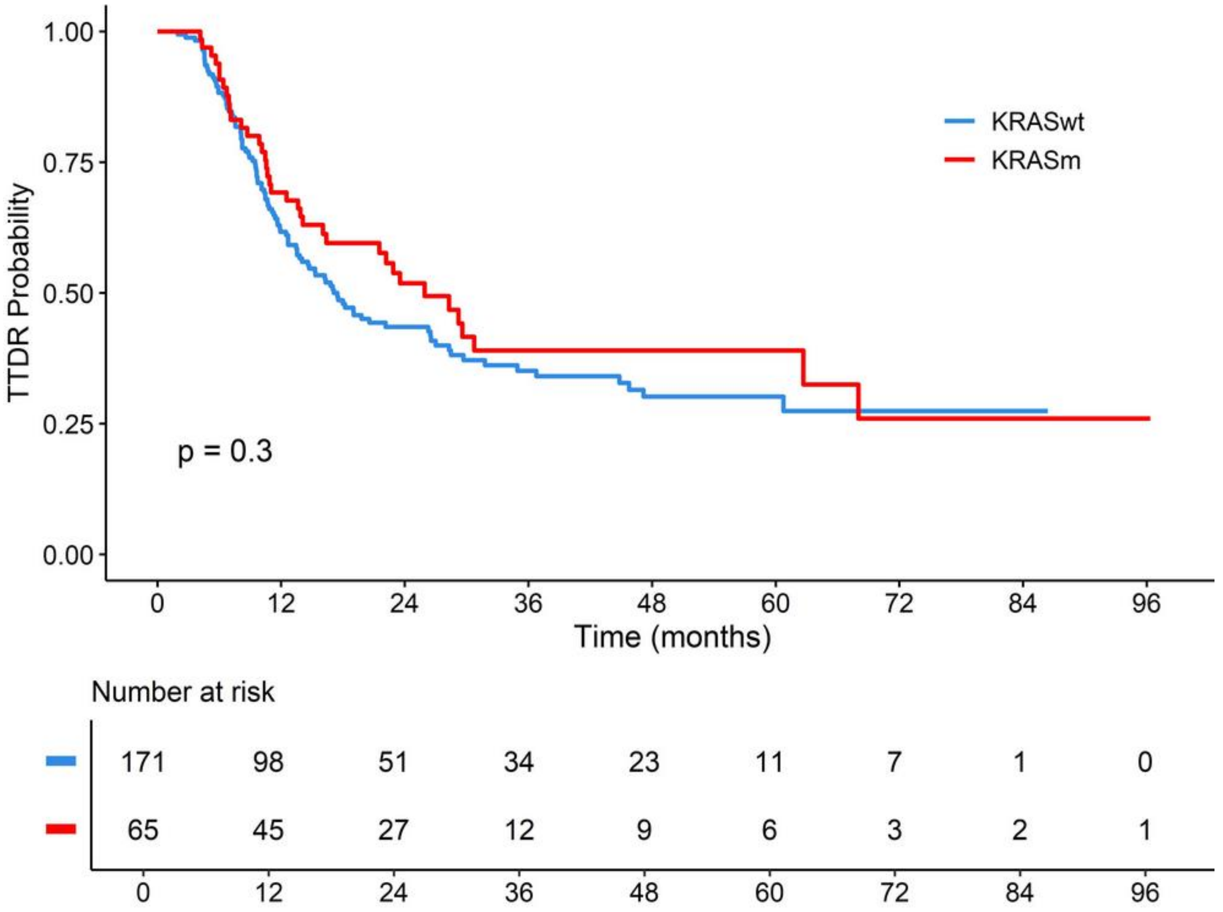

KRASwt = KRAS wild-type; KRASm= KRAS mutation; TTDR = time to distant relapse; p-value considered statistically significant was less than 0.05.

Supplementary Figure 6 - Prognostic factors associated with TTLR in multivariate analysis

TTLR

	HR (95% CI)	p-value
**Age**	0.99 (0.98-1.01)	0.30
**WHO status**	-	-
0	-	-
1	1.39 (0.96-2.02)	0.08
2	1.96 (0.93-4.14)	0.08
**Type of CT**	-	-
Sequential	-	-
Concomitant	0.74 (0.52-1.07)	0.11
**Durvalumab**	-	-
No	-	-
Yes	0.39 (0.25-0.59)	<0.001
**KRAS mutation**	-	-
No	-	-
Yes	0.86 (0.61-1.22)	0.40

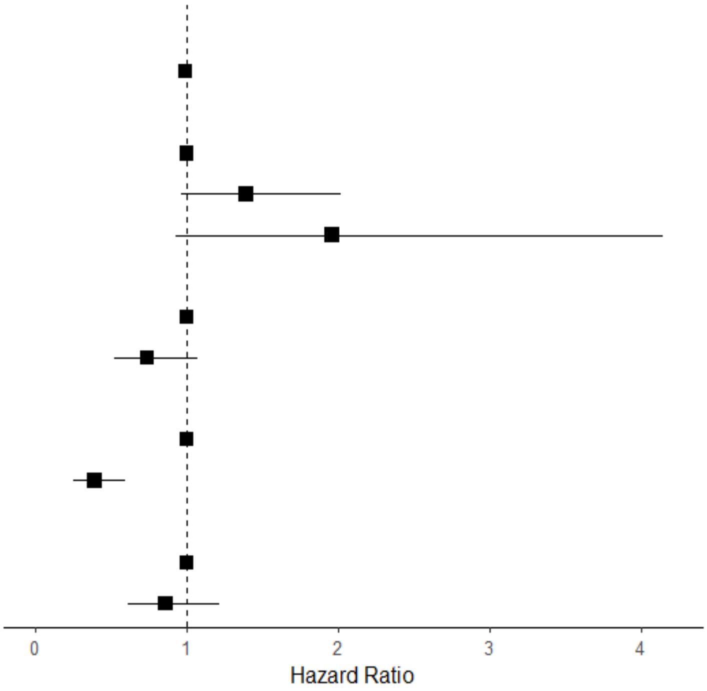

TTLR= time to local relapse; WHO status = World Health Organization status; N platine = number of platinum salt cycles; HR= hazard ratio; CT = chemoradiotherapy; p-value considered statistically significant was less than 0.05.

Supplementary Figure 7 - Prognostic factors associated with TTDR in multivariate analysis

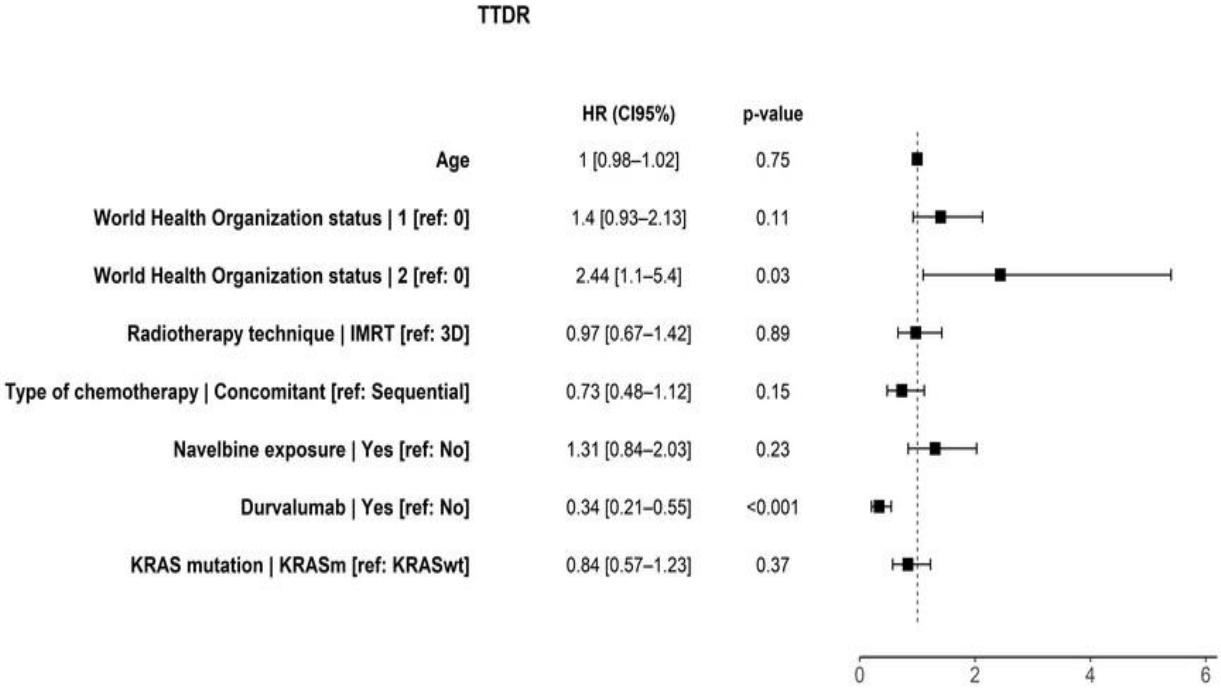

TTDR= time to distant relapse; WHO status = World Health Organization status; N platine = number of platinum salt cycles; HR= hazard ratio; IMRT = intensity-modulated radiation therapy; CT = chemoradiotherapy; *KRASm* = *KRAS* mutation; p-value considered statistically significant was less than 0.05.

Supplementary Figure 8 – Subgroup analysis for OS according to *KRASm*: durvalumab (a) and without durvalumab (b)

(a)

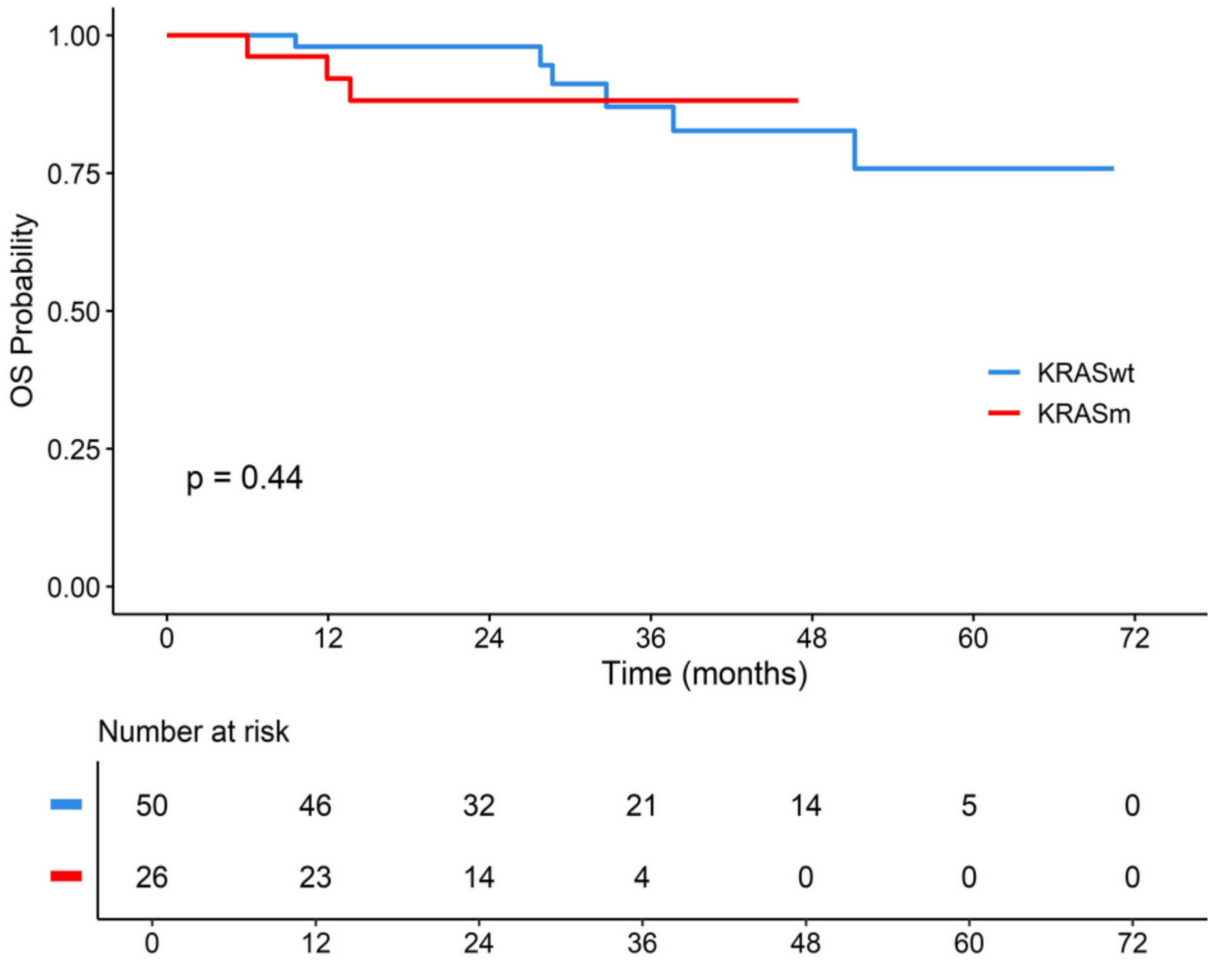

OS = overall survival; KRASwt = KRAS wild-type; KRASm= KRAS mutation; p-value considered statistically significant was less than 0.05.

(b)

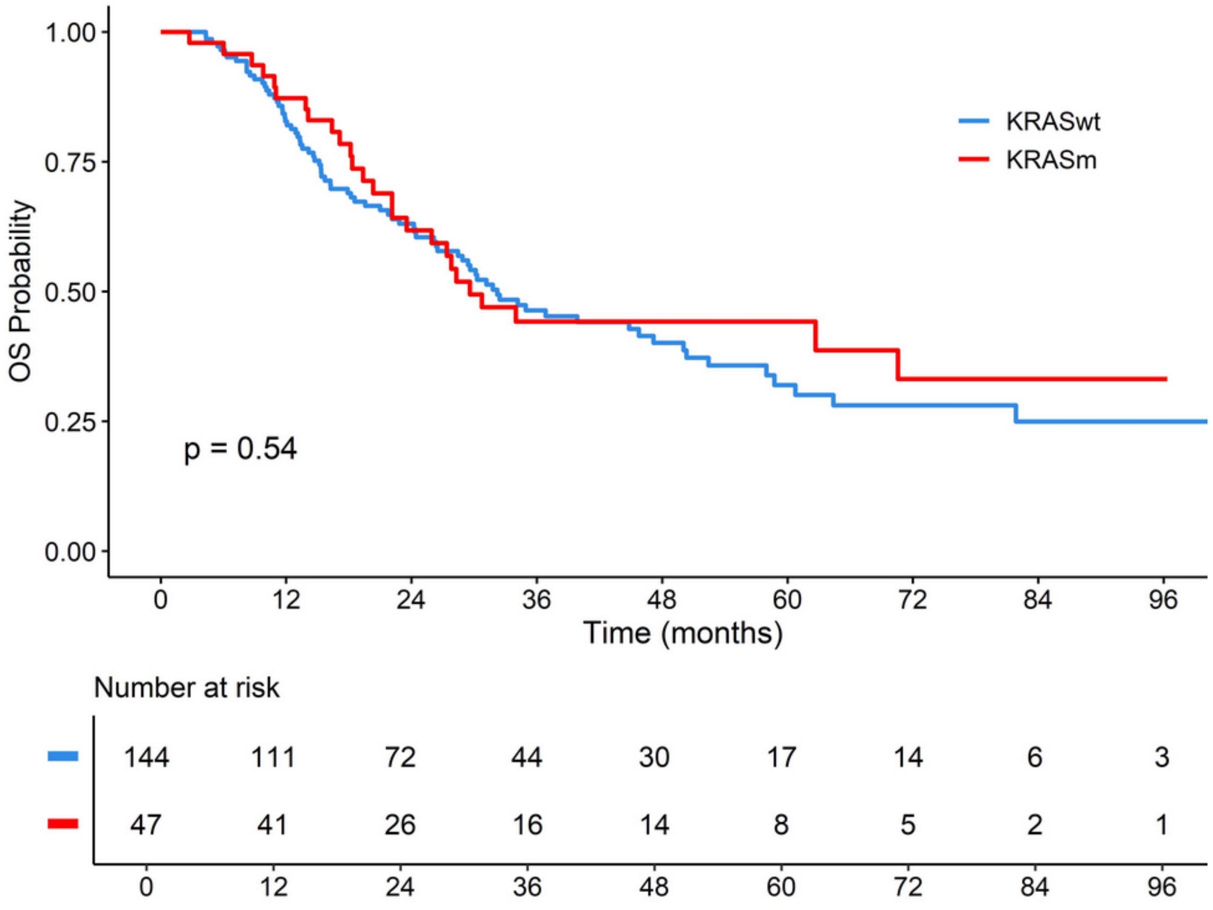

OS = overall survival; KRASwt = KRAS wild-type; KRASm= KRAS mutation; p-value considered statistically significant was less than 0.05.

Supplementary Figure 9 – Subgroup analysis for PFS according to *KRASm* : durvalumab (a) and without durvalumab (b)

(a)

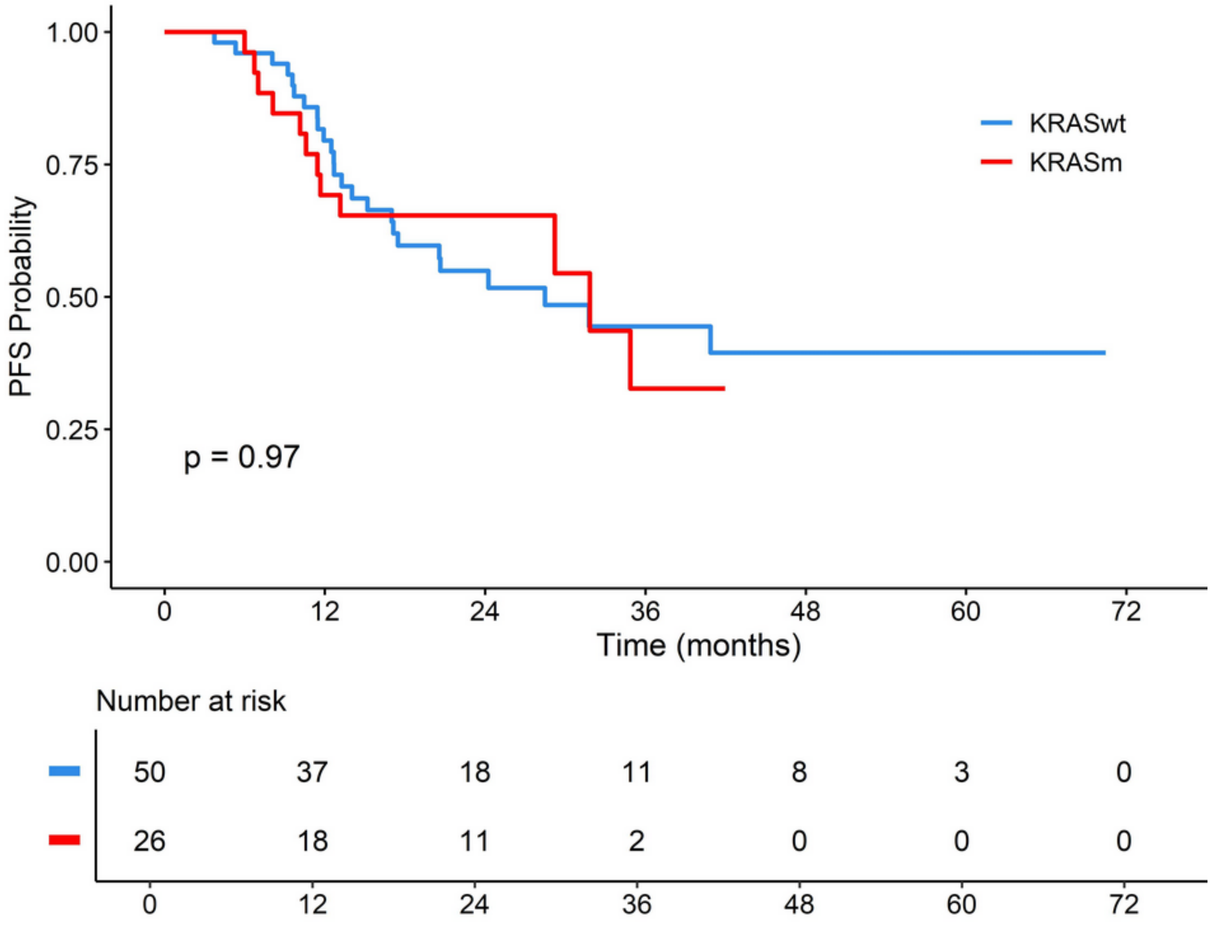

PFS = progression-free survival; KRASwt = KRAS wild-type; KRASm= KRAS mutation; p-value considered statistically significant was less than 0.05.

(b)

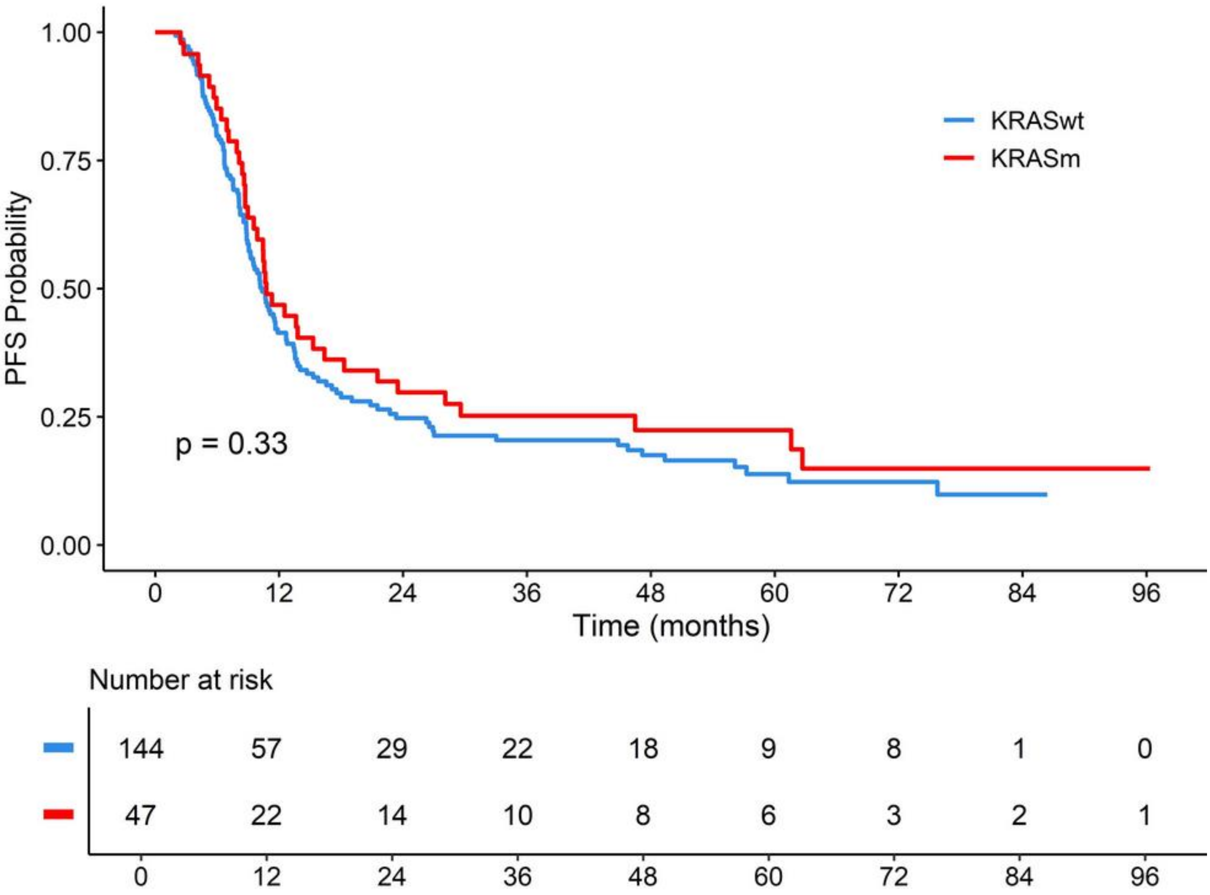

PFS = progression-free survival KRASwt = KRAS wild-type; KRASm= KRAS mutation; p-value considered statistically significant was less than 0.05.

Supplementary Figure 10 - Subgroup analysis for TTLR according to *KRASm*: durvalumab (a) and without durvalumab (b)

(a)

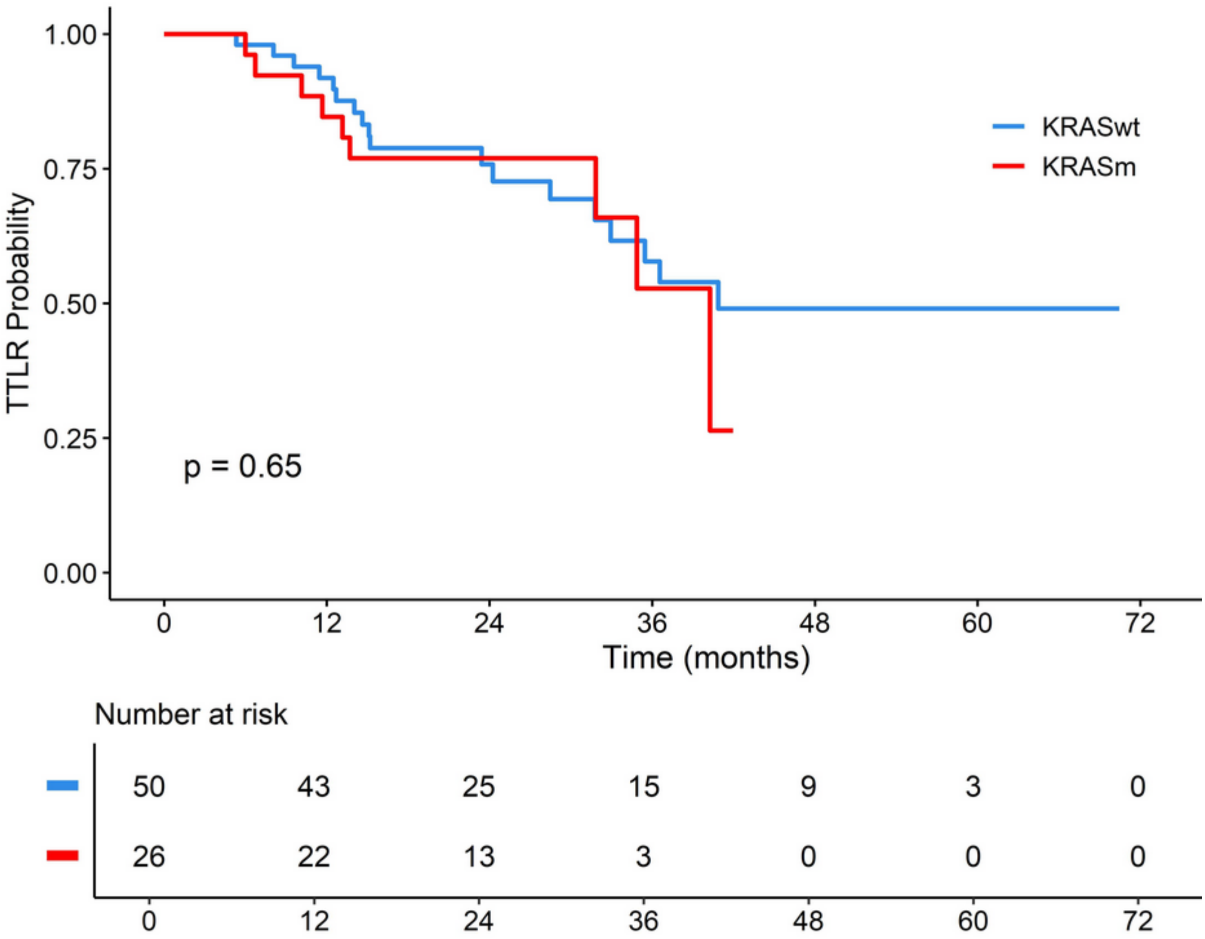

KRASwt = KRAS wild-type; KRASm= KRAS mutation; TTLR = time to local relapse; p-value considered statistically significant was less than 0.05.

(b)

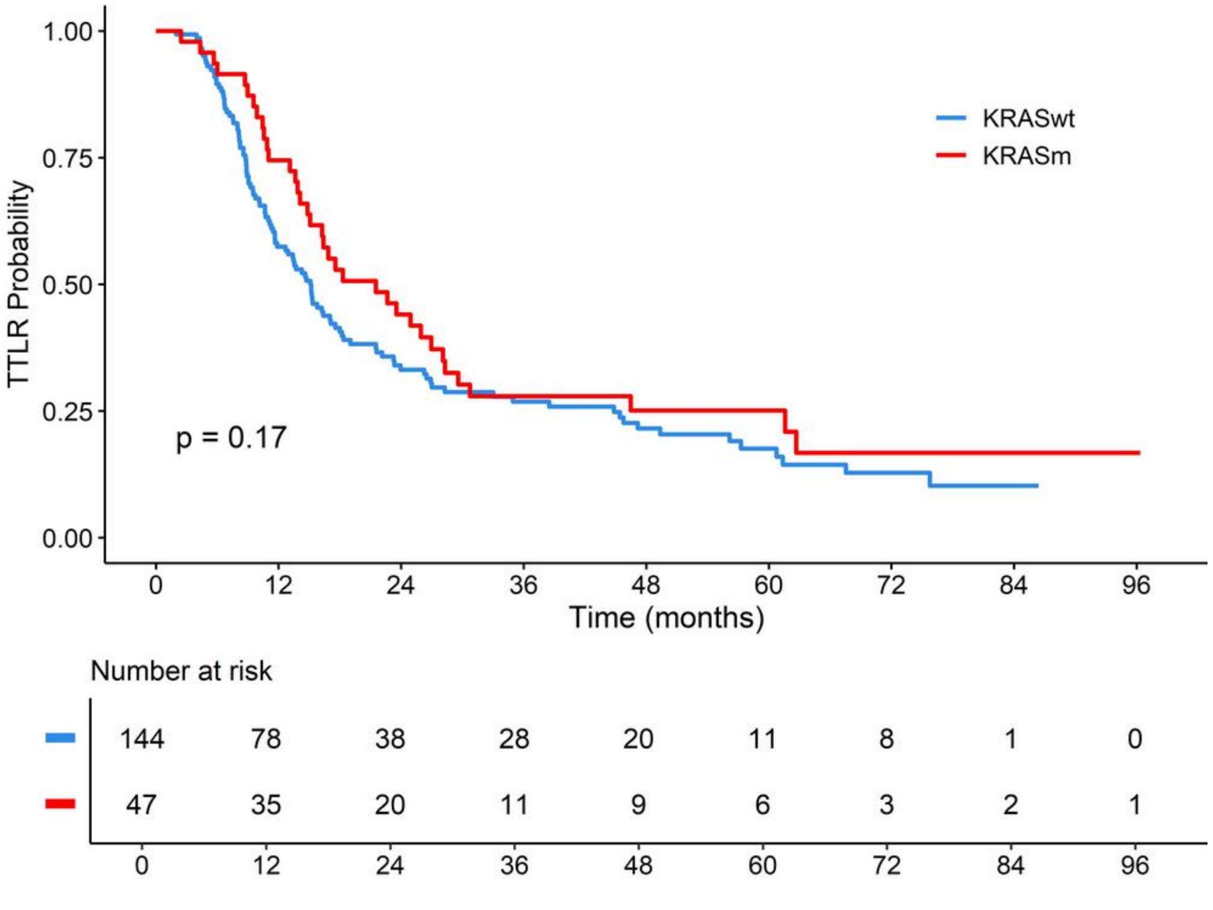

KRASwt = KRAS wild-type; KRASm= KRAS mutation; TTLR = time to local relapse; p-value considered statistically significant was less than 0.05.

Supplementary Figure 11 - Subgroup analysis for TTDR according to *KRASm*: durvalumab (a) and without durvalumab (b)

(a)

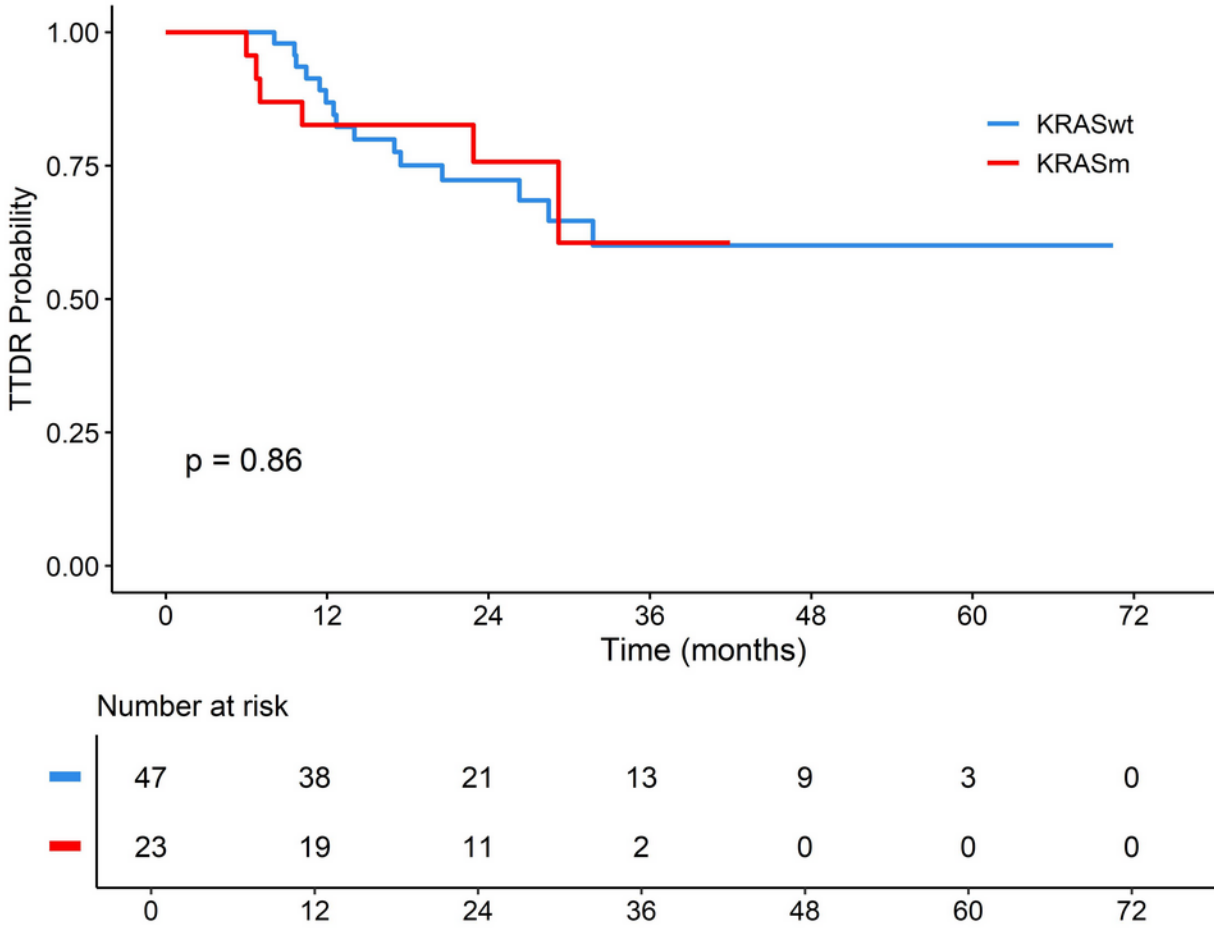

KRASwt = KRAS wild-type; KRASm= KRAS mutation; TTDR= time to distant relapse; p-value considered statistically significant was less than 0.05.

(b)

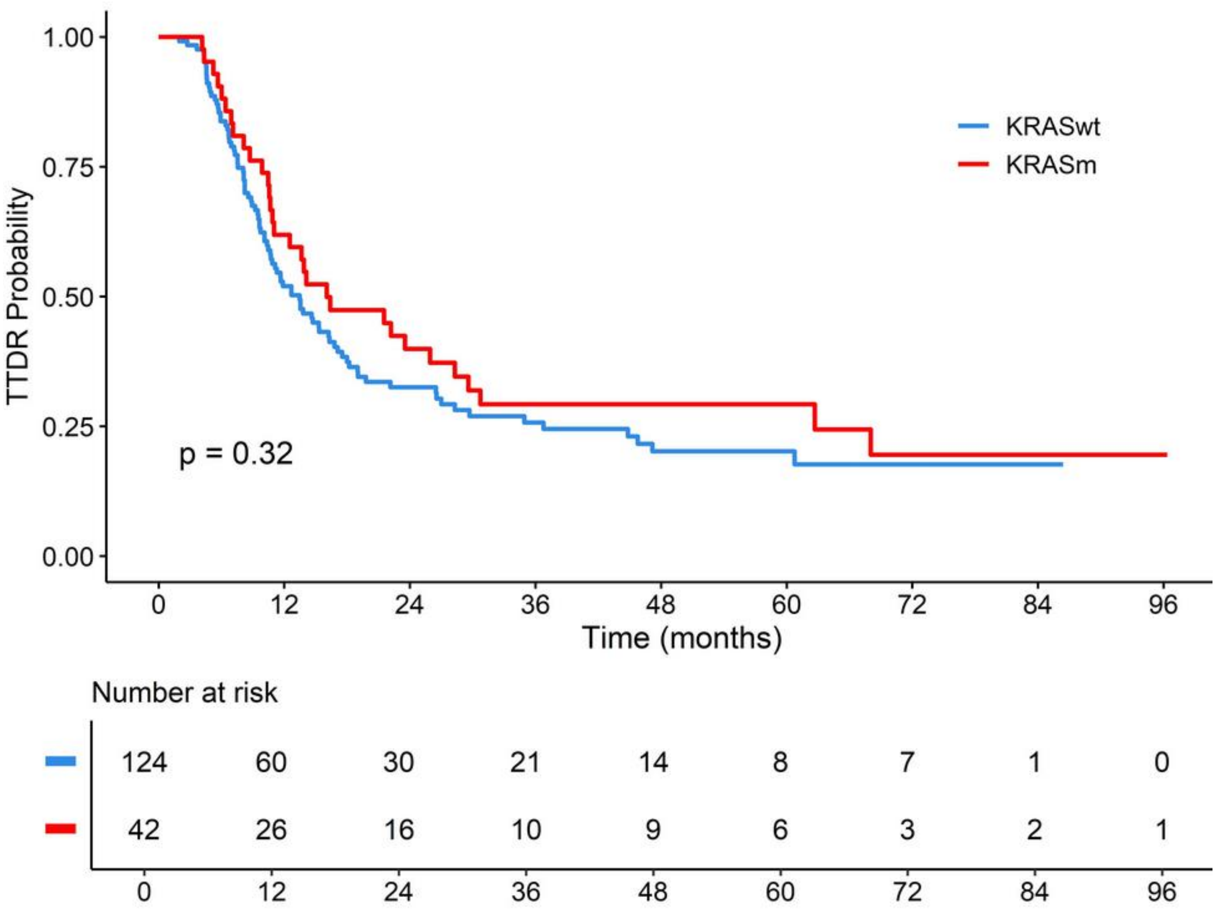

KRASwt = KRAS wild-type; KRASm= KRAS mutation; TTDR= time to distant relapse; p-value considered statistically significant was less than 0.05.

Supplementary Figure 12 – Subgroup analysis for OS according to durvalumab treatment

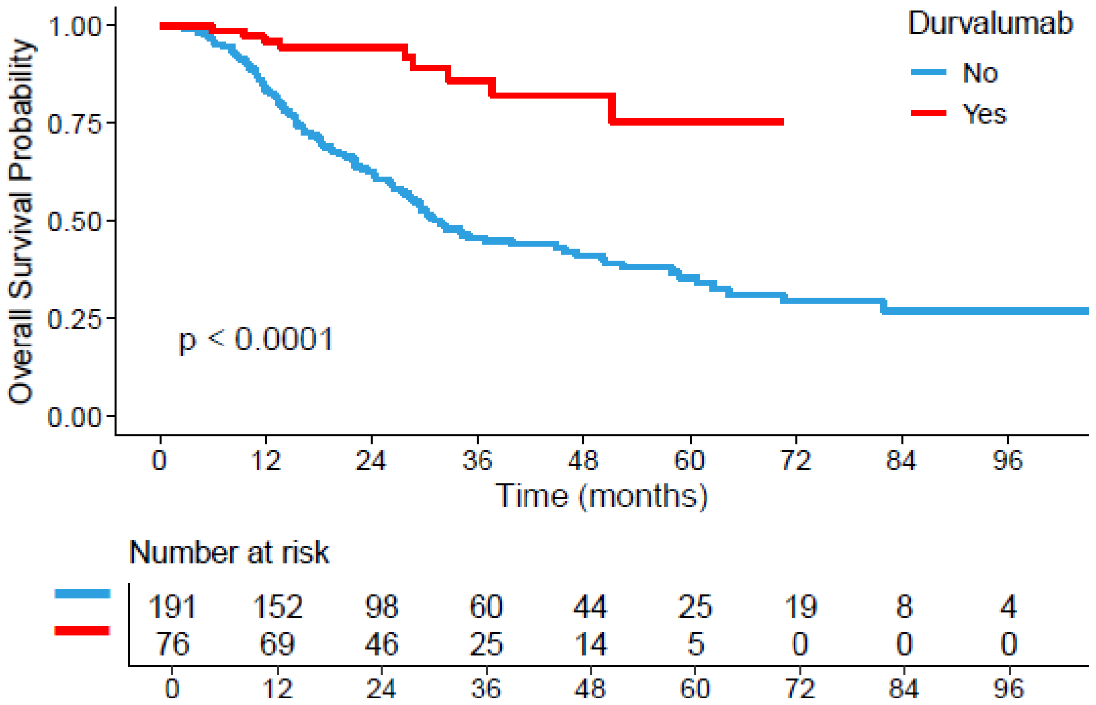

OS = overall survival; p-value considered statistically significant was less than 0.05.

Supplementary Figure 13 – Subgroup analysis for PFS according to durvalumab treatment

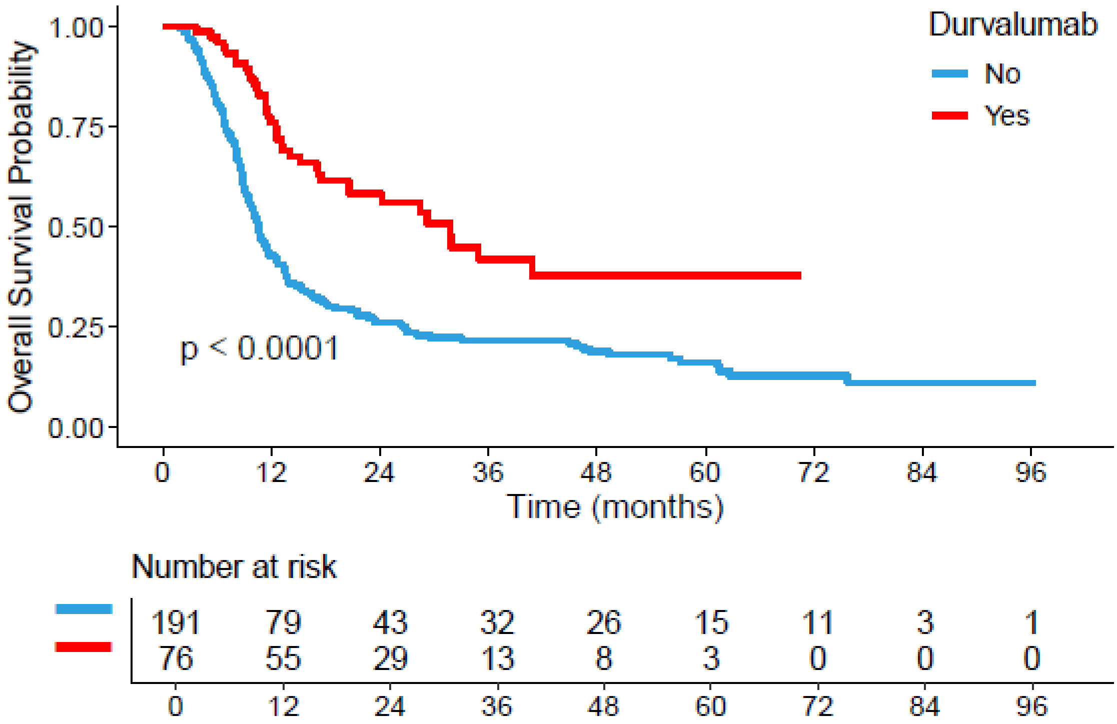

PFS = progression-free survival; p-value considered statistically significant was less than 0.05.

Supp.Table 14 - Comparison of *KP* and *KPwt* for response to chemoradiotherapy

	KP	*KPwt*	Total	p.
	(N=20)	(N=46)	(N=66)	
ORR				0.92
No	11(55%)	23 (50%)	34(52%)	
Yes	9 (45%)	23 (40%)	32(48%)	
DCR				0.092
No	0(0%)	7 (15%)	7(11%)	
Yes	20 (100%)	39 (85%)	59 (89%)	

KP = *KRAS/TP53 comutation*; *KPwt* = Patients without *KRAS* or *TP53* mutations; ORR = objective response rate; DCR = disease control rate; p-value considered statistically significant was less than 0.05.

Supp.Table 15 - OS comparison between *KP and KPwt*

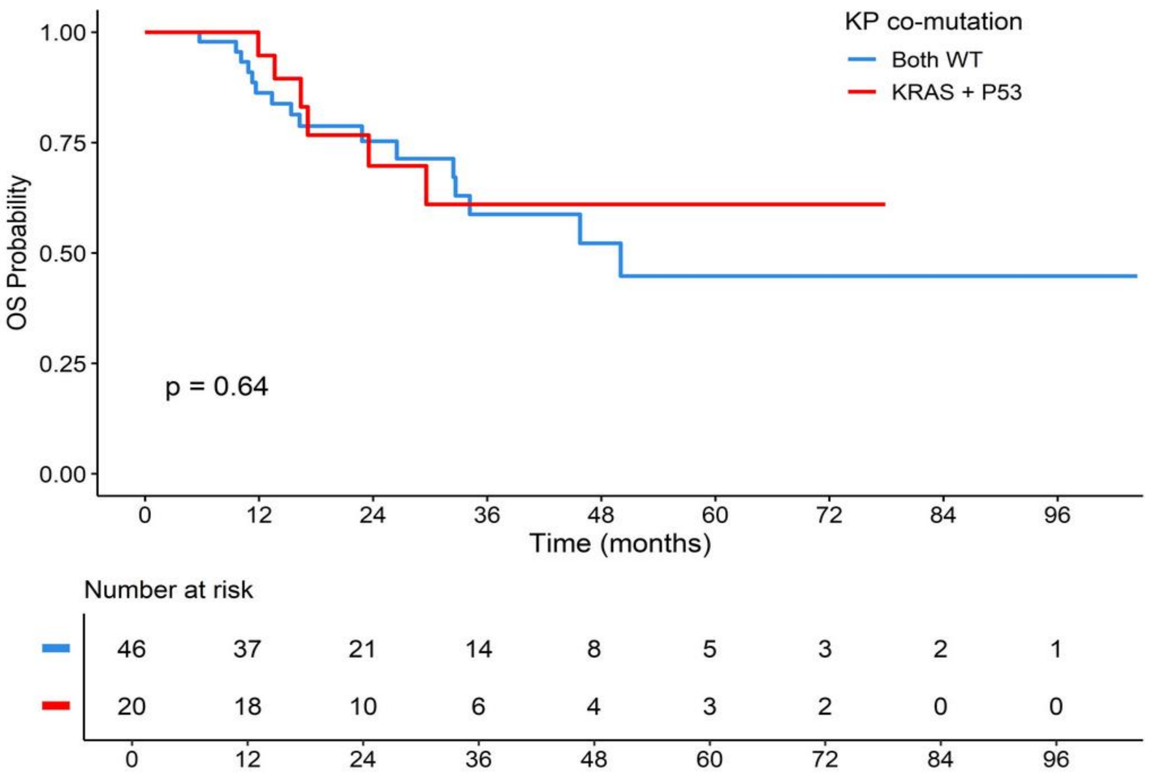

KP = *KRAS/TP53 comutation*; *KPwt* = Patients without *KRAS* or *TP53* mutations; *KPwt* = both wild-type; WT = wild-type; OS = overall survival; p-value considered statistically significant was less than 0.05.

Supp.Table 16 - PFS comparison between *KP and KPwt*

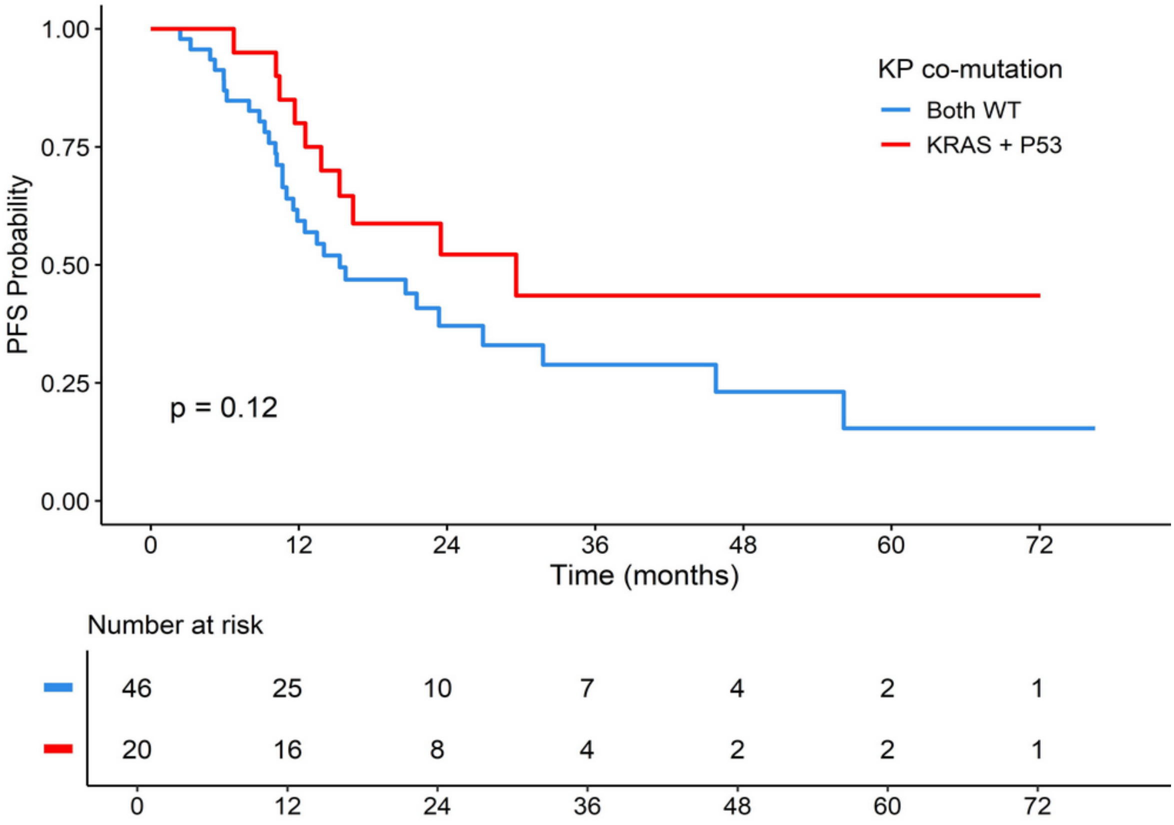

KP = KRAS/TP53 comutation; *KPwt* = Patients without *KRAS* or *TP53* mutations; *KPwt* = both wild-type; WT = wild-type; PFS = progression-free survival; p-value considered statistically significant was less than 0.05.

Supp.Table 17 - Comparison of *KL* and *KLwt* for response to chemoradiotherapy

	KL	*KLwt*	Total	p.
	(N=4)	(N=152)	(N=156)	
ORR				0.62
No	1(25%)	76 (50%)	77(49%)	
Yes	3 (75%)	76 (40%)	79(51%)	
DCR				1
No	0(0%)	21 (14%)	21(13%)	
Yes	4 (100%)	131 (86%)	135 (87%)	

KL = *KRAS/STK11 comutation*; *KLwt* = Patients without *KRAS* or *STK11* mutations.; ORR = objective response rate; DCR = disease control rate; p-value considered statistically significant was less than 0.05.

Supp.Table 18 - OS comparison between *KL and KLwt*

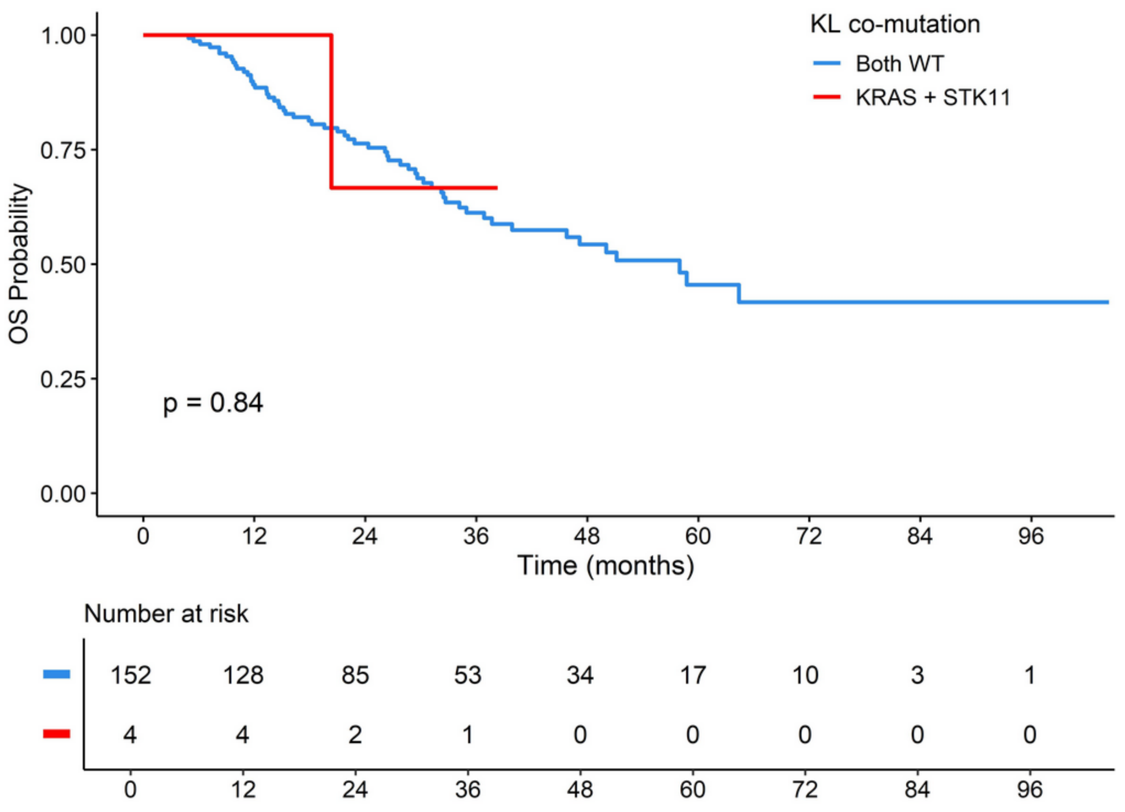

KL = *KRAS/STK11 comutation*; *KLwt* = Patients without *KRAS* or *STK11* mutations; *KLwt* = both wild-type; WT = wild-type; OS = overall survival; p-value considered statistically significant was less than 0.05.

Supp.Table 19 - PFS comparison between *KL and KLwt*

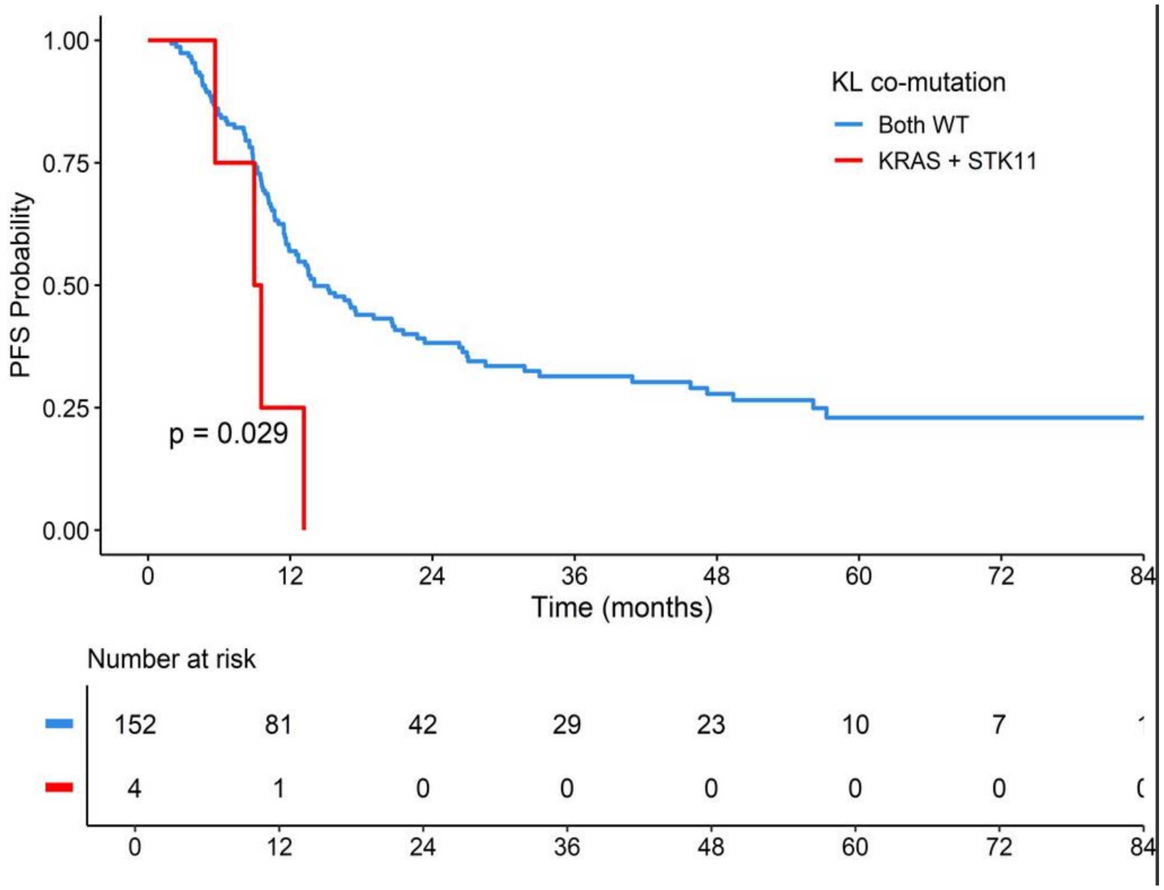

KL = *KRAS/STK11 comutation*; *KLwt* = Patients without *KRAS* or *STK11* mutations; *KLwt* = both wild-type; WT = wild-type; PFS = progression-free survival; p-value considered statistically significant was less than 0.05.

Supp.Table 20 - results of the univariate analysis on OS, PFS, TTLR and TTDR

		OS		PFS		TTLR	
**Characteristic**	**N**	**HR (95% CI)**	**p-value**	**HR (95% CI)**	**p-value**	**HR (95% CI)**	**p-value**
Age	267	1.00 (0.98, 1.02)	0.77	1.00 (0.98, 1.01)	0.73	1.00 (0.98, 1.01)	0.90
Sex	267	-	-	-	-	-	-
Female [ref: male]	-	0.78 (0.52, 1.17)	0.23	1.22 (0.91, 1.63)	0.19	1.18 (0.86, 1.62)	0.30
PS	267	-	-	-	-	-	-
1 [ref: 0]	-	**1.75 (1.09, 2.81)**	**0.021**	1.27 (0.90, 1.79)	0.18	1.31 (0.91, 1.90)	0.15
2 [ref: 0]	-	**3.54 (1.51, 8.32)**	**0.004**	**2.81 (1.36, 5.82)**	**0.005**	**2.20 (1.06, 4.57)**	**0.034**
Tobacco	258	-	-	-	-	-	-
Never [ref: Active+Weaned]	-	0.92 (0.40, 2.09)	0.84	1.23 (0.67, 2.27)	0.50	1.37 (0.74, 2.53)	0.31
Stage	253	-	-	-	-	-	-
Stage IIIA [ref: II]	-	1.48 (0.66, 3.28)	0.34	1.14 (0.65, 1.99)	0.64	1.01 (0.56, 1.80)	0.98
Stage IIIB [ref: II]	-	1.65 (0.75, 3.66)	0.22	1.27 (0.73, 2.21)	0.40	1.12 (0.63, 1.99)	0.69
Stage IIIC [ref: II]	-	**2.49 (1.00, 6.20)**	**0.050**	**2.04 (1.06, 3.93)**	**0.032**	1.66 (0.83, 3.32)	0.15
History of lung cancer	267	-	-	-	-	-	-
Yes [ref: No]	-	1.45 (0.73, 2.88)	0.28	0.95 (0.54, 1.67)	0.85	1.13 (0.61, 2.08)	0.70
History of lung cancer surgery	267	-	-	-	-	-	-
Yes [ref: No]	-	1.08 (0.55, 2.13)	0.83	0.74 (0.42, 1.30)	0.29	0.82 (0.44, 1.51)	0.52
RT technique	267	-	-	-	-	-	-
IMRT [ref: 3D]	-	0.84 (0.56, 1.25)	0.38	0.77 (0.56, 1.05)	0.10	0.80 (0.57, 1.11)	0.18
Duration of the RT	267	1.03 (1.00, 1.06)	0.062	1.01 (0.98, 1.04)	0.52	1.01 (0.98, 1.04)	0.57
RT dose	266	0.94 (0.86, 1.04)	0.25	0.96 (0.89, 1.04)	0.34	0.97 (0.90, 1.05)	0.50
Type of CRT	267	-	-	-	-	-	-
Concomitant [ref: Sequential]	-	**0.57 (0.38, 0.85)**	**0.006**	**0.69 (0.50, 0.94)**	**0.020**	**0.64 (0.46, 0.89)**	**0.009**
Platinium salts	180	-	-	-	-	-	-
Carboplatine	-	-	-	-	-	-	-
Cisplatine [ref: Carboplatine]	-	1.16 (0.70, 1.91)	0.56	1.16 (0.78, 1.73)	0.47	1.09 (0.72, 1.65)	0.68
Both [ref: Carboplatine]	-	0.75 (0.10, 5.43)	0.77	0.75 (0.18, 3.06)	0.69	0.83 (0.20, 3.39)	0.79
Number of platinium salts	267	**0.74 (0.62, 0.88)**	**<0.001**	0.88 (0.78, 1.00)	0.059	0.91 (0.79, 1.04)	0.17
Taxane exposure	267	-	-	-	-	-	-
Yes [ref: No]	-	1.06 (0.74, 1.54)	0.74	0.93 (0.70, 1.23)	0.6	0.92 (0.68, 1.24)	0.58
Pemetrexed exposure	267	-	-	-	-	-	-
Yes [ref: No]	-	**0.67 (0.46, 0.98)**	**0.039**	0.84 (0.63, 1.11)	0.22	0.89 (0.66, 1.20)	0.44
Navelbine exposure	267	-	-	-	-	-	-
Yes [ref: No]	-	1.19 (0.76, 1.87)		**1.48 (1.02, 2.14)**	**0.037**	1.34 (0.92, 1.95)	0.13

PDL1	183	-	-	-	-	-	-
<=50% [ref: No]	-	0.75 (0.41, 1.36)	0.34	0.85 (0.57, 1.27)	0.44	0.86 (0.55, 1.33)	0.50
>50% [ref: No]	-	0.65 (0.33, 1.29)	0.22	**0.59 (0.37, 0.96)**	**0.032**	0.65 (0.39, 1.08)	0.094
Durvalumab	267	-	-	-	-	-	-
Yes [ref: No]	-	**0.21 (0.10, 0.41)**	**<0.001**	**0.43 (0.30, 0.61)**	**<0.001**	**0.37 (0.24, 0.56)**	**<0.001**
KRAS G12C mutation	267	-	-	-	-	-	-
Mutated [ref: Wild-type]	-	0.93 (0.55, 1.56)	0.78	1.03 (0.70, 1.50)	0.90	0.92 (0.61, 1.37)	0.67
EFGR mutation	266	-	-	-	-	-	-
Mutated [ref: Wild-type]	-	1.35 (0.66, 2.78)	0.41	1.39 (0.77, 2.50)	0.27	1.51 (0.82, 2.79)	0.19
BRAF mutation	265	-	-	-	-	-	-
Mutated [ref: Wild-type]	-	0.72 (0.32, 1.64)	0.43	0.89 (0.50, 1.60)	0.70	1.26 (0.70, 2.26)	0.45
P53 mutation	167	-	-	-	-	-	-
Mutated [ref: Wild-type]	-	1.42 (0.86, 2.34)	0.18	1.05 (0.72, 1.53)	0.80	0.94 (0.63, 1.41)	0.76
STK11 mutation	219	-	-	-	-	-	-
Mutated [ref: Wild-type]	-	1.47 (0.59, 3.65)	0.40	**3.53 (2.00, 6.23)**	**<0.001**	1.79 (0.91, 3.53)	0.094
ALK mutation	266	-	-	-	-	-	-
Mutated [ref: Wild-type]	-	0.32 (0.05, 2.32)	0.26	0.88 (0.33, 2.37)	0.80	0.67 (0.21, 2.11)	0.50
RET mutation	237	-	-	-	-	-	-
Mutated [ref: Wild-type]	-	2.43 (0.34, 17.6)	0.38	0.79 (0.11, 5.62)	0.81	1.02 (0.14, 7.28)	0.99
KRAS/STK11 co-mutation	156	-	-	-	-	-	-
KRAS + STK11 [ref: Both wild-type]	-	0.82 (0.11, 5.91)	0.84	**2.92 (1.06, 8.05)**	**0.038**	**5.07 (1.81, 14.2)**	**0.002**
KRAS/P53 co-mutation	66	-	-	-	-	-	-
KRAS + P53 [ref: Both wild-type]	-	0.80 (0.31, 2.05)	0.65	0.57 (0.28, 1.17)	0.13	0.55 (0.25, 1.22)	0.14

OS = overall survival; PFS= progression-free survival; TTLR = time to local relapse; TTDR = time to distant relapse; *KRAS* = kirsten rat sarcoma ; ; OS = overall survival; PS = performance status; HR = hazard ratio; IMRT = intensity-modulated radiation therapy; CRT = chemoradiotherapy ; PD-L1 = programmed death-ligand 1; *EGFR* = epithelial growth factor receptor; *BRAF* = v-Raf murine sarcoma viral oncogene homolog B; *ALK* = anaplastic lymphoma kinase; *STK11* = serine/threonine kinase 11; *RET* = rearranged during transfection; *TP53* = tumor protein 53; p-value considered statistically significant was less than 0.05.
